# A Combined Phenotypic and Metabolomic Approach for Elucidating the Biostimulant Action of a Plant-Derived Protein Hydrolysate on Tomato Grown Under Limited Water Availability

**DOI:** 10.3389/fpls.2019.00493

**Published:** 2019-05-03

**Authors:** Kenny Paul, Mirella Sorrentino, Luigi Lucini, Youssef Rouphael, Mariateresa Cardarelli, Paolo Bonini, Maria Begoña Miras Moreno, Hélène Reynaud, Renaud Canaguier, Martin Trtílek, Klára Panzarová, Giuseppe Colla

**Affiliations:** ^1^Photon Systems Instruments, spol. s.r.o., Drásov, Czechia; ^2^Department for Sustainable Food Process, Research Centre for Nutrigenomics and Proteomics, Università Cattolica del Sacro Cuore, Piacenza, Italy; ^3^Department of Agricultural Sciences, University of Naples Federico II, Portici, Italy; ^4^Consiglio per la Ricerca in Agricoltura e l’Analisi dell’Economia Agraria, Centro di Ricerca Orticoltura e Florovivaismo, Pontecagnano Faiano, Italy; ^5^NGAlab, Tarragona, Spain; ^6^Italpollina USA, Inc., Anderson, IN, United States; ^7^Nixe, Valbonne, France; ^8^Department of Agriculture and Forest Sciences, Tuscia University, Viterbo, Italy; ^9^Arcadia Srl, Rivoli Veronese, Italy

**Keywords:** protein hydrolysates, high-throughput phenotyping, metabolomics, morpho-physiological traits, foliar spray, drench application

## Abstract

Plant-derived protein hydrolysates (PHs) are an important category of biostimulants able to increase plant growth and crop yield especially under environmental stress conditions. PHs can be applied as foliar spray or soil drench. Foliar spray is generally applied to achieve a relatively short-term response, whereas soil drench is used when a long-term effect is desired. The aim of the study was to elucidate the biostimulant action of PH application method (foliar spray or substrate drench) on morpho-physiological traits and metabolic profile of tomato grown under limited water availability. An untreated control was also included. A high-throughput image-based phenotyping (HTP) approach was used to non-destructively monitor the crop response under limited water availability (40% of container capacity) in a controlled environment. Moreover, metabolic profile of leaves was determined at the end of the trial. Dry biomass of shoots at the end of the trial was significantly correlated with number of green pixels (*R*^2^ = 0.90) and projected shoot area, respectively. Both drench and foliar treatments had a positive impact on the digital biomass compared to control while the photosynthetic performance of the plants was slightly influenced by treatments. Overall drench application under limited water availability more positively influenced biomass accumulation and metabolic profile than foliar application. Significantly higher transpiration use efficiency was observed with PH-drench applications indicating better stomatal conductance. The mass-spectrometry based metabolomic analysis allowed the identification of distinct biochemical signatures in PH-treated plants. Metabolomic changes involved a wide and organized range of biochemical processes that included, among others, phytohormones (notably a decrease in cytokinins and an accumulation of salicylates) and lipids (including membrane lipids, sterols, and terpenes). From a general perspective, treated tomato plants exhibited an improved tolerance to reactive oxygen species (ROS)-mediated oxidative imbalance. Such capability to cope with oxidative stress might have resulted from a coordinated action of signaling compounds (salicylic acid and hydroxycinnamic amides), radical scavengers such as carotenoids and prenyl quinones, as well as a reduced biosynthesis of tetrapyrrole coproporphyrins.

## Introduction

Competition among agriculture, industry, and cities for limited water supplies is already constraining development efforts in many countries. As populations expand and economies grow, the competition for limited supplies will intensify and so will conflicts among water users. Agriculture is not only the world’s largest water user in terms of volume; it is also a relatively low-value, low-efficiency, and highly subsidized water user (Rouphael et al., 2012).

These facts are forcing farmers to grow crops with diminishing water supplies. Limited water availability can affect morphological, physiological, biochemical, and molecular processes in plants, resulting in growth depression and yield reduction (Liu et al., 2014; Kumar et al., 2017). Under these conditions, the application of plant biostimulants can help crops to use water more efficiently by changing the root-to-shoot ratio, plant metabolism, and hormonal balance (Colla et al., 2017b; Rouphael and Colla, 2018).

Protein hydrolysates (PHs) represent an important category of plant biostimulants that have been extensively used for improving crop yield and quality especially under abiotic stress conditions such as limited water, salinity, and heavy metals (Ertani et al., 2009; Colla et al., 2015; du Jardin, 2015). PHs could directly stimulate carbon and nitrogen metabolism and could indirectly enhance nutrient availability of substrates and increase nutrient uptake as well as nutrient-use efficiency in plants (Haplern et al., 2015; Colla et al., 2017b; Rouphael et al., 2017). PHs can be applied by foliar spray or substrate drench, affecting molecular and physiological crop response in a different way (Lucini et al., 2015; Sestili et al., 2018). In a recent study, substrate drench applications of a plant-derived PH were more effective to improve plant growth and total N uptake than foliar sprays in tomato (Sestili et al., 2018). In the same study, the application method (drench or foliar) of the plant-derived PH affected the expression of genes encoding ammonium and nitrate transporters differently as well as seven enzymes involved in N metabolism of tomato (Sestili et al., 2018). Biostimulant activity of PH can be due to the direct effect of bioactive compounds (e.g., signaling peptides, free amino acids) on plant metabolism and to the indirect effect resulting from the PH-mediated enhancement of plant growth promoting microorganisms in plant microbiome (Luziatelli et al., 2019).

A successful evaluation of biostimulant activity of PHs requires an accurate measurement of morpho-physiological traits of plants over time. Use of advanced image-based automated phenotyping platforms offers opportunities to increase both the speed at which these measurements are collected and the accuracy of measurements (Povero et al., 2016). Dynamic screening of plants can be done for multiple morpho-physiological traits related to growth, yield, and performance throughout their development or onset, progression, and recovery from abiotic stress (Petrozza et al., 2014). Functional action and characterization of PHs in plants can be thus monitored with high precision and in high resolution in each phase of plant development and/or plant response to environmental conditions, depending on the target substance application or type of experimental layout (Rouphael et al., 2018b). Range of morpho-physiological traits can be monitored in a fully automated, high-resolution, and high-sensitivity manner. A key descriptive parameter in plant physiology, except for root analysis, is the shoot growth of the plants. Quantitative and qualitative dynamic assessment of growth performance by RGB imaging was used to characterize range of traits such as shoot biomass or yield (Li et al., 2014; Humplík et al., 2015b). Non-invasive monitoring of plant photosynthetic activity is also critical for understanding the physiological and metabolic condition, as well as its susceptibility to various stress conditions (Gorbe and Calatayud, 2012; Humplík et al., 2015a; Paul et al., 2016). Pulse-amplitude-modulation-based kinetic chlorophyll fluorescence imaging is a broadly applied technique used to understand the plant phenology in response to external stimuli or agents (Murchie and Lawson, 2013). In a high-throughput phenotyping setup, modern imaging systems were recently successfully used to monitor dynamically PSII parameters and electron flow dynamics at the whole plant level (Humplík et al., 2015b; Awlia et al., 2016; Tschiersch et al., 2017). Usage of automated photosynthetic phenotyping approaches helps us to screen and characterize PH real-time interaction throughout the grow regime. Water taken up by plants or plant water content is key for understanding the efficiency with which plants are able to regulate stomatal conductance and CO_2_ fixation. Water content in plants is the result of the equilibrium between root water uptake and shoot transpiration (Berger et al., 2010). Thermoimaging has been used in high-throughput phenotyping platforms to monitor plant transpiration rate and transpiration use efficiency (TUE) (Kaňa and Vass, 2008; Paul et al., 2016).

In addition to dynamic screening of plant performance by automated plant phenotyping, metabolomics offers unique opportunities to understand the mode of action of PHs on crops and to identify biomarkers of biostimulant action. For instance, Lucini et al. (2015) identified several differentially expressed key metabolites associated with osmotic adjustment, oxidative stress mitigation, and hormone network in PH-treated lettuce plants exposed to salt stress. Considering that tomato is among the most important crops grown in the world, an experimental trial was performed to evaluate the biostimulant activity of a plant-derived PH applied through foliar spray or substrate drench on tomato plants grown under limited water availability in a controlled environment. The research phases of the trial included (1) the use of a high-throughput phenotyping platform for evaluating the treatment effects on selected morpho-physiological traits of plants (e.g., digital biomass, kinetic chlorophyll fluorescence and leaf surface temperature) and (2) the use of mass-spectrometry (MS) based metabolomics for identifying distinct biochemical signatures in PH-treated plants (including hormones and secondary metabolites produced by plants in response to low water availability stress conditions).

## Materials and Methods

### Plant Material and Growing Conditions

Seeds of tomato (*Solanum lycopersicum* L.–Hybrid F1 Chicco Rosso) were sown in trays with size of pots of 100 ml each containing a commercial peat-based substrate (Substrate 2, Klasmann-Deilmann GmbH, Germany) having the following characteristics: density, 160 kg m^3^; total pore space, 85% v/v; total carbon, 55%; pH 5.5; N, 210 mg L^−1^; P, 105 mg L^−1^; K, 224 mg L^−1^; and Mg, 100 mg L^−1^; trace elements in chelated forms. Substrate was watered to water holding capacity. Trays with seeds were kept for 2 days at 4°C in the dark. Trays with seeds were placed in the controlled growth chamber (FS-WI, PSI, Czechia) at a 16-h day/8-h night regime, 22°C day/20°C night, 60% relative humidity, and with cool-white LED (250 μmol photons m^−2^ s^−1^) and far-red LED (5.5 μmol photons m^−2^ s^−1^) lighting.

### Fertigation and Watering Protocol

Prior to plant transplanting into 3-L pots, trays were uniformly watered at 6, 7, 12, and 14 days after placement of trays in a controlled growth chamber. On day 7 and day 14, plants were fertigated with a solution containing 1.04 g L^−1^ calcium nitrate (15.5% N; 28% CaO), 0.04 g L^−1^ ammonium nitrate (34% N), 0.14 g L^−1^ monopotassium phosphate (52% P_2_O_5_, 34% K_2_O), 0.18 g L^−1^ potassium sulfate (50% K_2_O, 45% SO_3_), 0.5 g L^−1^ magnesium sulfate (10% N, 16% MgO), and 0.5 ml L^−1^ FloraMicro (5% N, 1% K_2_O, 5% Ca, 0.01% B, 0.001% Cu, 0.1% Fe, 0.05% Mn, 0.0008% Mo, and 0.015% Zn).

Twenty-day-old plants were selected with uniform growth characteristics and transplanted into 3-L pots (mixture of Substrate 2 Klasmann soil and river sand in 3:1 ratio was used). The pots were labeled with unique identification codes for each plant replicate and treatment. For determining the water content at container capacity, one set of substrate pots was dried for 3 days at 80°C and another set was saturated with water and left to drain for 1 day before weighing 100% water holding capacity (Awlia et al., 2016). Water content at container capacity was calculated as the difference between substrate weight at water holding capacity and dried substrate. On the day before transplantation, soil was prepared, and moisture content was adjusted to 60% of container capacity. Twenty-one-day-old plants were transplanted into the prepared substrate mixture with 60% of container capacity. Following the transplantation, plants were regularly watered to reference weight (40% of container capacity) defined as low water availability condition by using the automated watering and weighing unit of the PlantScreen^TM^ Modular System (Photon Systems Instruments (PSI), Czechia).

### Biostimulant Characteristics

Plant-derived PH biostimulant Trainer^®^ was provided by Italpollina Company (Rivoli Veronese, Italy). The plant-derived PH Trainer^®^ is a commercial PH obtained through enzymatic hydrolysis of proteins derived from legume seeds. Briefly, the seeds are ground, and the flour was dispersed in acidified water to extract the soluble compounds. Filtration and centrifugation are then used to separate the protein concentrate from the other organic compounds. Enzymatic hydrolysis is used to release the amino acids and peptides from protein concentrate. Insoluble residual compounds are separated from amino acids and peptides by centrifugation. The resulting PH is concentrated through water evaporation (Colantoni et al., 2017). The final product contains mostly peptides and amino acids and, with a less extent, soluble carbohydrates, mineral elements and phenolic compounds. Trainer^®^ has a density of 1.21 kg L^−1^, a dry matter of 46%, and a pH of 4.0. It contains 310 g kg^−1^ of free amino acids and soluble peptides (Rouphael et al., 2018a). The aminogram of the product (in g kg^−1^) was as follows: Ala (12), Arg (19), Asp (33), Cys (4), Glu (54), Gly (13), His (8), Ile (12), Leu (24), Lys (19), Met (4), Phe (16), Pro (15), Ser (17), Thr (11), Trp (4), Tyr (13), and Val (16). The antioxidant activity of Trainer^®^, as measured by ferric-reducing antioxidant power (FRAP), was 41.9 mmol Fe^2+^ g^−1^ f.w., while the total phenolics and flavonoids, determined following the methods reported by Borgognone et al. (2014), were 8.93 mg of gallic acid equivalent per gram of f.w. product and 0.95 mg of quercetin equivalent per gram of f.w. product, respectively. The Trainer^®^ content of soluble sugars was 90 g kg^−1^ f.w., and its elemental composition was as follows (g kg^−1^ f.w): N (50.0), P (0.9), K (41.1), Ca (10.9), Mg (0.5), Fe (0.024), Zn (0.010), Mn (0.001), B (0.005), and Cu (0.001) (Colla et al., 2017a). The Trainer^®^ content of N–NO_3_ and N–NH_4_ was 3.13 and 6.00 μg g^−1^ f.w., respectively (Ceccarelli, 2018). No detectable phytohormones in Trainer^®^ have been reported (Luziatelli et al., 2019).

### Plant Identification and Biostimulant Treatments

Plants were randomly distributed into three groups with six biological replicates per group. Three groups each containing six plants were identified as follows: no application, foliar application, and drench application of PH. Each plant was labeled with a unique barcode identifier used for registration of the plants in the PlantScreen^TM^ Modular System.

The PH was applied either as foliar spray or as substrate drench (Supplementary Figure S1B) as water solution containing a non-ionic surfactant Triton X-100 at 0.1%. A control group (no application) was sprayed with distilled water containing 0.1% Triton X-100. PH application was performed twice: 5 days after transplanting (DAT) referred to as Treatment 1 (T1) and 12 DAT referred to as Treatment 2 (T2). For 24 h prior to and following spraying, humidity in the cultivation chamber was kept at 85% relative humidity. For foliar spray treatments, 2 ml of PH was diluted in 500 ml of distilled water with 0.1% Triton X-100, and 60 ml of solution was applied by homogeneous foliar spray over the entire plant surface per plant replica. Substrate of each pot was covered with aluminum foil during and upon spraying and was removed prior to the next phenotypical analysis in the PlantScreen^TM^ Modular System. For drenching treatment, 4 ml of biostimulant was diluted in 1,000 ml of 0.1% Triton, and 60 ml per plant replicate was applied by drenching. At both PH application times (T1 and T2), plants in control treatment and those foliarly sprayed with PH were irrigated with 60 ml of water each to avoid changes of substrate water status in comparison with plants treated by drench application of PH. Right after PH treatment, plants were taken back to fytoscope FS-WI.

### High-Throughput Plant Phenotyping Protocol and Imaging Sensors

Plant phenotypic measurements were performed using the PlantScreen^TM^ Modular System installed in semi-controlled greenhouse environment conditions in the PSI Research Center (PSI, Drásov, Czechia). The platform was operated in closed imaging loop located in a climatized environment with temperature ranging between 21°C and 24°C. The platform is equipped with four robotic-assisted imaging units, an automatic height measuring light curtain unit, an acclimation tunnel, and a weighing and watering unit. Plants placed in individual transportation disks were transported by moving belt toward individual imaging units and watering and weighing stations.

Twenty-two-day-old plants were randomly distributed into three batches, each batch containing 12 plants. Plant imaging started with 22-day-old plants (1 DAT, day 1 of phenotyping) and continued for 15 days (15 DAT, day 15 of phenotyping). Plants were imaged using the following protocol. Briefly, plants were manually transferred from the climate-controlled growth chamber to the manual loading station of the PlantScreen^TM^ Modular System and were transported through the acclimation tunnel with automatic height measuring unit. Prior to the imaging, plants were dark-adapted in acclimation tunnel for 15 min. Each batch of plants was automatically phenotyped for around 30 min by using kinetic chlorophyll fluorescence imaging measurement for photosynthetic performance analysis; top view and multiple-angle side view Red Green Blue (RGB) imaging for morphological, growth, and color analysis; and finally a thermal imaging unit for plant surface temperature quantification (Supplementary Figure S1A). Following the imaging, plants were automatically transported to the watering and weighting unit for maintaining precise soil water holding capacity. After completion of the phenotyping protocol, plants were manually moved back to the climate-controlled growth chamber until the subsequent phenotyping day. We used the automatic timing function of the PlantScreen^TM^ Scheduler (PSI, Czechia) to schedule the initiation of the phenotyping protocol at the same time of the diurnal cycle (after 3 h of illumination in the climate-controlled growth chamber). The phenotyping data were acquired twice prior to biostimulant application in days 1 and 3 (pre-T measurements), three times post-first biostimulant application in days 6, 8, and 10 (post-T1 application), and twice post-second biostimulant application in days 13 and 15 (post-T2 application). The acquired images were automatically processed using Plant Data Analyzer (PSI, Czechia), and the raw data exported into CSV files were provided as input for further analysis.

### Kinetic Chlorophyll Fluorescence Measurement

Kinetic chlorophyll fluorescence (ChlF) measurements were acquired using an enhanced version of the FluorCam FC-800MF pulse amplitude modulated (PAM) chlorophyll fluorometer (PSI, Czechia) with an imaging area in top view position of 800 × 800 mm, as described in Tschiersch et al. (2017). We assessed the photosynthetic performance in the plants by quantifying the rate of photosynthesis at different photon irradiances using the light curve protocol (Henley, 1993; Rascher et al., 2000). The measuring protocol described previously (Awlia et al., 2016) was optimized for the tomato plants from early to later developmental stage. For the light curve characterization, three actinic light irradiances (Lss1–170 μmol photons m^−2^ s^−1^, Lss2–620 μmol photons m^−2^ s^−1^, and Lss3–1,070 μmol photons m^−2^ s^−1^) were used with a duration of 30 s in order to quantify the rate of photosynthesis.

From the fluorescence data, a range of parameters were extracted as described in detail by Awlia et al. (2016). Additionally, 1 - *q_P_* was calculated, which reflects the proportion of PSII reaction centers that are closed (Maxwell and Johnson, 2000; Na et al., 2014).

### Visible Red Green Blue Imaging

To assess digital biomass of the plants, RGB imaging was done from top view (RGB2) and side view from multiple angles (RGB1). The RGB imaging unit is a light-isolated box equipped with turning table with precise angle positioning and two RGB cameras (top and side) mounted on robotic arms, each supplemented with LED-based lighting source to ensure homogeneous illumination of the imaged object.

Projected shoot area (PSA) parameter, together with regularly determined weight of the plants, was used to estimate TUE. TUE was defined by the ratio of aboveground biomass produced per unit of water transpired and depends on the characteristics of the plants and on the environment where the plants grow (Al-Tamimi et al., 2016). TUE was estimated from transpiration defined by measures of water loss and growth from PSA by plant-specific pixel counts quantification.

### Thermal Imaging

To assess leaf surface temperature of the plants, a thermal imaging unit based on side view imaging was used. The thermal imaging unit incorporated in the PlantScreen^TM^ System consists of a light-isolated box with one side view camera mounted on a robotic arm, precise plant positioning, and a background heated wall with an integrated temperature sensor to increase contrast for the image processing step. The imaged area is 1,205 × 1,005 mm (height × width). To assess spatio-temporal variations in temperature over plant surface, we used FLIR A615 thermal camera with 45° lens and resolution 640 × 710 pixels, with high-speed infrared windowing option and <50 mK thermal sensitivity (FLIR Systems Inc., Boston, MA, United States). The thermal images were acquired in line scan mode with each image consisting of 710 pixels with a scanning speed of 50 Hz (lines per second). Thermal images were acquired in darkness. Image acquisition conditions, plant positioning, and camera settings were fixed throughout the experiment. Leaf surface temperature of each plant was automatically extracted with Plant Data Analyzer software (PSI, Czechia) by mask application, background subtraction, and pixel-by-pixel integration of values across the entire plant surface area. To minimize the influence of the environmental variability and the difference in the image acquisition timing among individual plants, the raw temperature of each plant (°C) was normalized by the actual background temperature and expressed as Δ*T* (°C).

### Sample Harvest and Metabolomic Analysis

Plant material was harvested 19 DAT for metabolomic analysis by harvesting and combining the third and fourth fully expanded leaves from the top of each plant. Additionally, the final biomass of each plant was determined by measuring fresh weight and dry weight of the remaining shoot.

Plant samples were homogenized in pestle and mortar using liquid nitrogen, and then an aliquot (1.0 g) was extracted in 10 ml of 0.1% HCOOH in 80% aqueous methanol using an Ultra-Turrax (Ika T-25, Staufen, Germany) (Borgognone et al., 2016). The extracts were centrifuged (12,000 × *g*) and filtered into amber vials through a 0.22-μm cellulose membrane for analysis. Thereafter, metabolomic analysis was carried out through a ultra-high performance liquid chromatograph (UHPLC) coupled to a quadrupole-time-of-flight mass spectrometer (UHPLC/QTOF-MS). The metabolomic facility included a 1290 ultra-high-performance liquid chromatograph, a G6550 iFunnel Q-TOF mass spectrometer, and a JetStream Dual Electrospray ionization source (all from Agilent Technologies, Santa Clara, CA, United States). The untargeted analysis was carried out as previously described (Rouphael et al., 2016). Briefly, reverse-phase chromatography was carried out on an Agilent Zorbax Eclipse-plus C18 column (100 × 2.1 mm, 1.8 μm) and using a 34-min linear elution gradient (5% to 95% methanol in water, with a flow of 220 μL min^−1^ at 35°C). The mass spectrometric acquisition was done in SCAN (100–1,000 *m/z*) and positive polarity (Pretali et al., 2016).

Features deconvolution and post-acquisition processing were done in Agilent Profinder B.06. Mass and retention time alignment followed by a filter-by-frequency postprocessing filter were done to retain only those compounds that were present in >75% of replications within at least one treatment. Compound annotation was done using the “find-by-formula” algorithm, i.e., using monoisotopic accurate mass, isotope spacing, and isotope ratio, with a mass accuracy tolerance of <5 ppm. The database PlantCyc 12.5 (Plant Metabolic Network^1^) was used for annotation purposes. Based on the strategy adopted, identification was carried out according to Level 2 (putatively annotated compounds) of the COSMOS Metabolomics Standards Initiative^2^. The classification of differential compounds into biochemical classes was carried out following PubChem (NCBI^3^) and PlantCyc information.

### Data Management and Statistical Analysis

For automatic image data processing, we used the data processing pipeline Plant Data Analyzer, which includes preprocessing, segmentation, feature extraction, and postprocessing of acquired images. Values for projected shoot area were calculated from images taken in the visible light spectrum and correspond to plant volume estimation. The plant volume was used as a proxy for the estimated biomass of the plants. Data were processed using MVApp application. Statistical differences between treatments and time points were determined by one-way analysis of variance (ANOVA) with *post hoc* Tukey’s Honest Significant Difference (HSD) test (*p*-value < 0.05) performed using appropriate scripts in MVApp tool. Data are displayed as mean ± standard error of the six independent plants per treatment.

Elaboration of metabolomic data was carried out using Mass Profiler Professional B.12.06 as previously described (Salehi et al., 2018). Briefly, compounds’ abundance was Log2 transformed and normalized at the 75th percentile and then baselined against the median. Unsupervised hierarchical cluster analysis was carried out using the fold-change-based heatmap, setting similarity measure as “Euclidean” and “Wards” linkage rule. Thereafter, the dataset was exported into SIMCA 13 (Umetrics, Malmö, Sweden), Pareto-scaled, and elaborated for Orthogonal Projections to Latent Structures Discriminant Analysis (OPLS-DA). This latter supervised statistic allowed the separation of variance into predictive and orthogonal (i.e., ascribable to technical and biological variation) components. Outliers were excluded using Hotelling’s T2 and adopting 95 and 99% confidence limits, for suspect and strong outliers, respectively. Model cross-validation was done through CV-ANOVA (*p* < 0.01), and permutation testing (*N* = 300) was used to exclude overfitting. Model parameters (goodness-of-fit *R*^2^*Y* and goodness-of-prediction *Q*^2^*Y*) were also produced. Finally, Variable Importance in Projection (VIP) analysis was used to select the metabolites having the highest discrimination potential. A subsequent fold-change analysis and two-way ANOVA were finally performed from VIPs to identify extent and direction of the changes in accumulation related to the use of the biostimulants.

Chemical Similarity Enrichment Analysis (Barupal and Fiehn, 2017) was finally performed on VIP metabolites to critically highlight the chemical nature of the discriminant compounds, as previously described (Showalter et al., 2018). Such enrichment analysis is based on chemical similarities and used Tanimoto substructure chemical similarity coefficients to cluster metabolites into non-overlapping chemical groups. In our elaborations, OPLS-DA VIP scores were used instead of individual *p*-values, and the regulation (up- or down-accumulation) of discriminant metabolites was compared across treatments following chemical enrichment. The online web-app tool^4^ was used for this analysis.

## Results

### Advanced Simultaneous Analysis of Morpho-Physiological Traits

**FIGURE 1:**
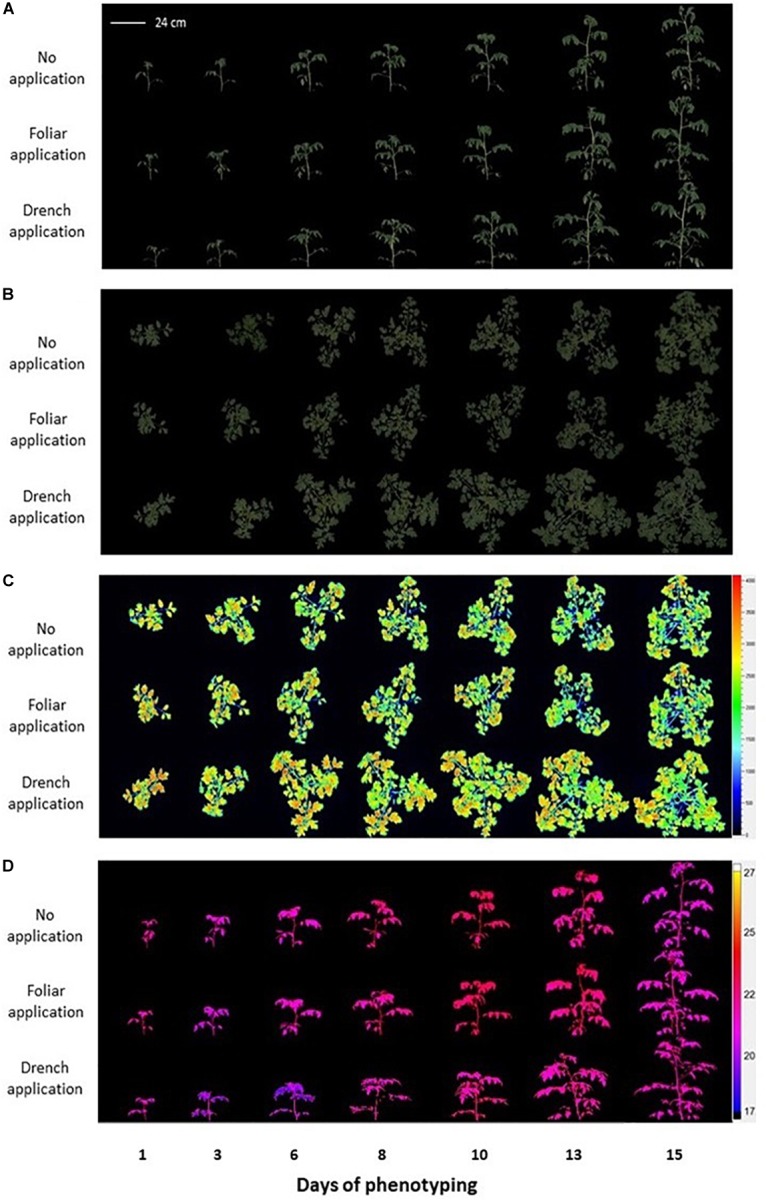
Non-invasive image-based phenotypical analysis of protein hydrolysate treated and control tomato plants grown under water-limiting conditions by using the PlantScreen^TM^ Modular System. **(A)** Color-segmented side view Red Green Blue (RGB) images of the tomato plants over the time of phenotyping period (D1–D15). **(B)** Color-segmented top view RGB images of the tomato plants. **(C)** False-color images of maximum fluorescence value (Fm) of tomato plants captured by kinetic chlorophyll fluorescence imaging. **(D)** False-color side view images of plant leaf surface temperature captured by thermal camera.

Integrative phenotyping facilities provide an opportunity to combine various methods of automated, simultaneous, non-destructive analyses for assessment of plant growth, morphology, and physiology. Here, we used the PlantScreen^TM^ Modular System (PSI, Czechia) available in the PSI Research Center (Drásov, Czechia) for simultaneous analysis of multiple morpho-physiological traits in tomato plants treated with plant-derived PH biostimulant substances (Supplementary Figure S1A). Tomato plants were cultivated under control conditions and were phenotyped by using RGB imaging to capture plant growth dynamics, morphology and color, by chlorophyll fluorescence (ChlF) imaging to quantify photosynthetic performance and by thermal imaging to analyze leaf surface temperature prior to and following the PH treatment (Figure 1). Finally, an automated watering and weighing unit was used to maintain constant low water availability conditions in the tomato plants treated with PH by both drenching and spraying applications (Supplementary Figure S1B).

### Visible Red Green Blue Imaging to Assess the Effect of Protein Hydrolysate on Plant Growth Dynamics

**FIGURE 2:**
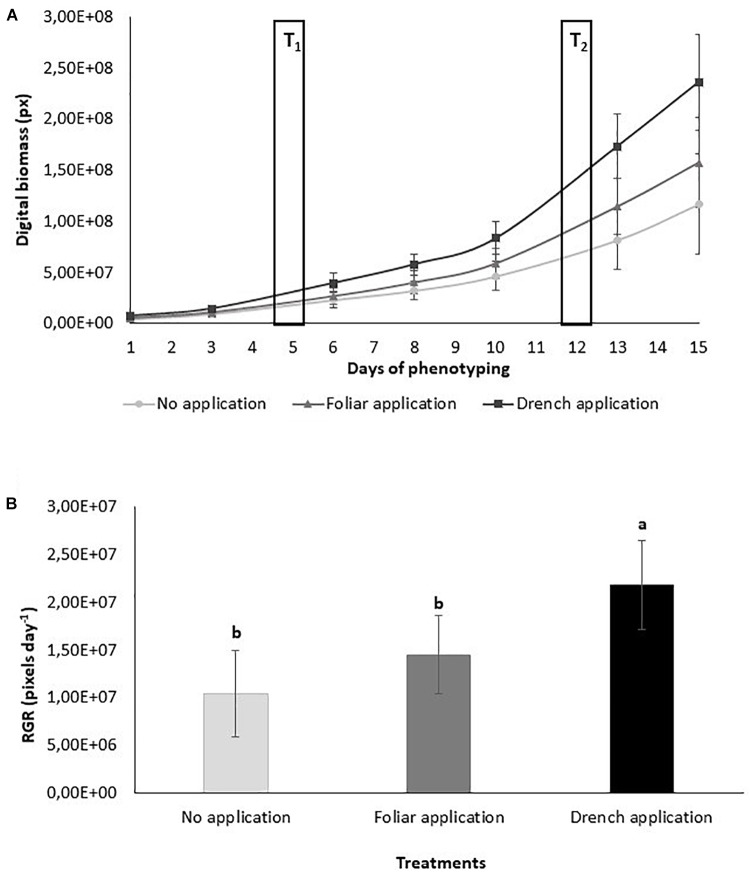
Growth performance of protein hydrolysate treated and control tomato plants. **(A)** Digital biomass quantified over time of phenotyping period. Values represent the average of six biological replicates per treatment. Error bars represent standard deviation. T1 and T2 correspond to days of protein hydrolysate application by foliar spraying or substrate drench. **(B)** Comparison of relative growth rate for the different treatments quantified over phenotyping period following the protein hydrolysate treatments. Values represent the average of six biological replicates per treatment. Error bars represent standard deviation. Different letters indicate significant difference according to one-way ANOVA *post hoc* Tukey’s test (*p* < 0.05).

Visible RGB digital color imaging was used for the assessment of range of visual traits in control plants (no application) and plants treated with PH by either drenching (drench application) or spraying application (foliar application) (Figure 1A,B). RGB imaging was used to quantify the effect of the PH on growth status, biomass accumulation, and color of tomato plants cultivated under limited water availability conditions (Figure 2A). Simple image stacks acquired from top view and two side view images were used to extract and calculate shoot volume as a proxy of shoot digital biomass and quantify shoot color throughout the cultivation period. The morphological traits were assed dynamically and were used to calculate growth rates (Figure 2B).

The analysis of the growth-related above-mentioned traits revealed that tomato plants cultivated under low water availability conditions and treated with PH by either spraying or drenching grew better than control plants. The best-performing plants treated with PH were those where PH was applied as substrate drench. At the end of the phenotyping period, the digital shoot biomass was significantly increased (Figure 2A and Supplementary Tables S1–S3) as well as the height and width of the plants (Supplementary Tables S4, S5). In addition, the growth rate calculated over the entire phenotyping period was also strongly enhanced in drench treated plants compared to foliarly sprayed ones under limited water availability (Figure 2B), suggesting that overall growth performance of the plants was improved following the drenching application of PH. The image-based data could be further confirmed by destructive plant biomass assessment as both fresh and dry weights of the PH-treated plants harvested at the end of the experiment were increased (Supplementary Figure S2A). Measurements of projected shoot area obtained using HTP imaging approach were strongly correlated with fresh and dry weights of the plants, and there was no indication of any deviation from a linear relationship even at the highest biomasses measured in this experiment (Supplementary Figures S2B,C).

The variation in shoot color of the tomato plants over the phenotyping period was assessed by quantification of greenness hue abundance from the color-segmented RGB images (Supplementary Figure S3). The analysis algorithms were calibrated by using RGB images from all treatments and all measurements as described previously (Awlia et al., 2016). Some minor changes were observed in the analyzed green hues, but no clear trend could be observed except for the slight increase in darker green hues at the end of the phenotyping period for the drench application variant (Supplementary Table S6).

### Mining the Biostimulant Action on Photosynthetic Performance

To assess the effect of PH application on photosynthetic performance of tomato plants under water-limiting conditions, chlorophyll fluorescence measurements were acquired using automated chlorophyll fluorescence imaging setup (Figure 1C and Supplementary Figure S1). The rate of photosynthesis at different photon irradiances was quantified using the light curve protocol reported by Henley (1993) and Rascher et al. (2000). From the measured fluorescence transient states, the basic ChlF parameters were derived (i.e., *F_o_*, *F*_m_, *F_t_*, and *F_v_*), which were used to calculate a range of parameters characterizing plant photosynthetic performance (i.e., *F_v_*/*F*_m_, NPQ, *q_P_*, and ΦPSII) [for an overview, refer to Paul et al. (2011); Awlia et al. (2016); Tschiersch et al. (2017)]. In addition, photochemical quenching (1 - *q_P_*) and photosynthetic electron transport rate (ETR) parameters were calculated, which refer to proportion of closed PSII reaction centers (Maxwell and Johnson, 2000) and ETR of photosystem II and indicate the efficiency of linear electron flow route in the photosynthetic machinery for producing energy-rich molecules adenosine triphosphate (ATP) and the reduced form of nicotinamide adenine dinucleotide phosphate (NADPH), respectively.

**FIGURE 3:**
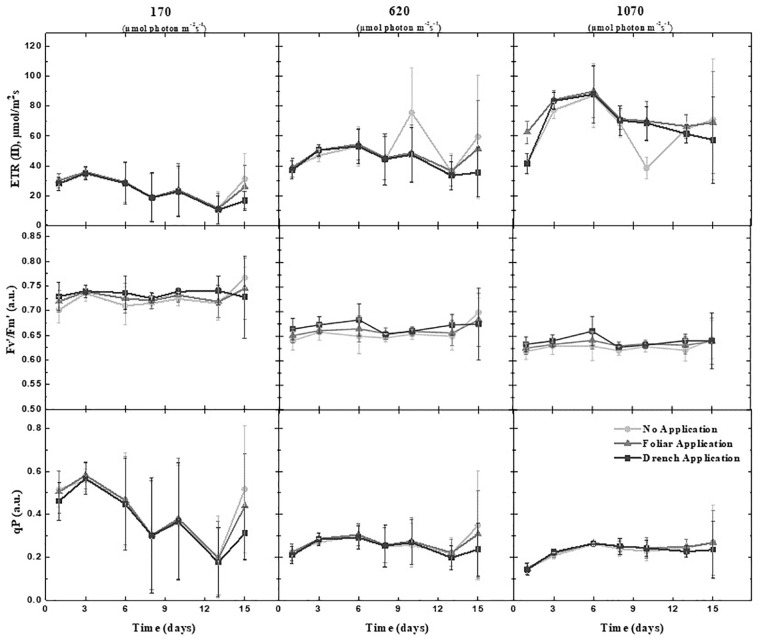
Photosynthetic performance of the tomato plants treated or untreated with protein hydrolysate. Range of photosynthetic parameters were deduced from kinetic chlorophyll fluorescence imaging prior to and following the PH treatments. The photochemical quenching coefficient that estimates the fraction of open PSII reaction centers (*q_P_*), maximum quantum yield of PSII photochemistry for the dark-adapted state (F_V_′/F_M_′), and electron transport rate (ETR) were measured using the light curve protocol. Data are mean of six independent plants per treatment. Measurements at three actinic photon irradiance intensities were acquired. Measurements were taken at 170, 620, and 1,070 μmol photons m^−2^ s^−1^, respectively.

A few of the parameters were selected to dynamically characterize the photosynthetic function of PSII in the tomato plants prior to and after the biostimulant treatment under limited water availability (Figure 3): the maximum quantum yield of PSII photochemistry in the dark-adapted state (*F_v_*/*F*_m_), the photochemical quenching coefficient that estimates the fraction of open PSII reaction centers (*q_P_*), steady-state non-photochemical quenching (NPQ), and ETR correlating to the quantum yield of the CO_2_ assimilation mechanisms and to the overall photosynthetic capacity of the plants (Genty et al., 1989). No significant changes of those parameters between the control and PH-treated plants (Figure 3 and Supplementary Table S7) were recorded during the phenotyping period. However, minor dynamic changes in lower actinic irradiance of the 1 - *q_P_* parameter were observed at the end of the phenotyping period on day 15 (Supplementary Figure S4). 1 - *q_P_* was used as an indicator of the closed PSII reaction center and as an estimate of the relative PSII excitation pressure to which an organism is exposed (Maxwell and Johnson, 2000), suggesting that PH application induced a higher redox status than control treatment, resulting in slightly lowered ETRs (Supplementary Figure S4).

### Thermal Infrared Imaging for Monitoring Shoot Temperature and Leaf Transpiration

Plant water status is determined by the equilibrium between root water uptake and shoot transpiration (Berger et al., 2010). Under limited water availability in tomato plants, triggering of shoot transpiration and root respiration has been carried out by commercial PH provided to the plant by foliar and drenching application, respectively. Imaging thermography approach was used to measure the whole plant temperature in an automated manner, and the image data were utilized to assess the leaf transpiration of plants (Figure 1D).

To minimize the influence of the environmental variability and the difference in the image acquisition timing among individual plants, the raw temperature of each plant (°C) was normalized by the actual background temperature and expressed as Δ*T* (°C) (Paul et al., 2016). Experimental data showed that leaf surface temperature of the tomato plants was not influenced by PH treatment, and no difference compared to control plants was observed throughout the entire phenotyping period (Supplementary Figure S5A). In addition to leaf surface temperature we assessed TUE that increased in drenching PH-treated plants in comparison with foliar and control treatments (Supplementary Figure S5B).

A strong correlation was reported between plant transpiration rate and stomatal conductance (Berger et al., 2010). As stomatal conductance is the measure of the CO_2_ entering or leaving the stomata of a leaf, higher TUE observed in PH-drench application might suggest that more CO_2_ might get fixed and generate more organic matter, thereby increasing in biomass compared to other treatment methods.

### Metabolomic Profiles

An untargeted UHPLC/QTOF-MS metabolomic analysis was carried out to elucidate the molecular mechanisms underlying the effect of PH application on leaves of tomato plants grown under limited water availability. Multivariate statistics from the metabolomic dataset pointed out similarities/dissimilarities among phytochemical profiles. The use of an untargeted profiling followed by annotation on the basis of a comprehensive database (namely, PlantCyc) produced over 1,900 compounds annotated, overall. These compounds exhibited a large chemical diversity and included metabolites from a wide range of biochemical classes and metabolic processes.

**FIGURE 4:**
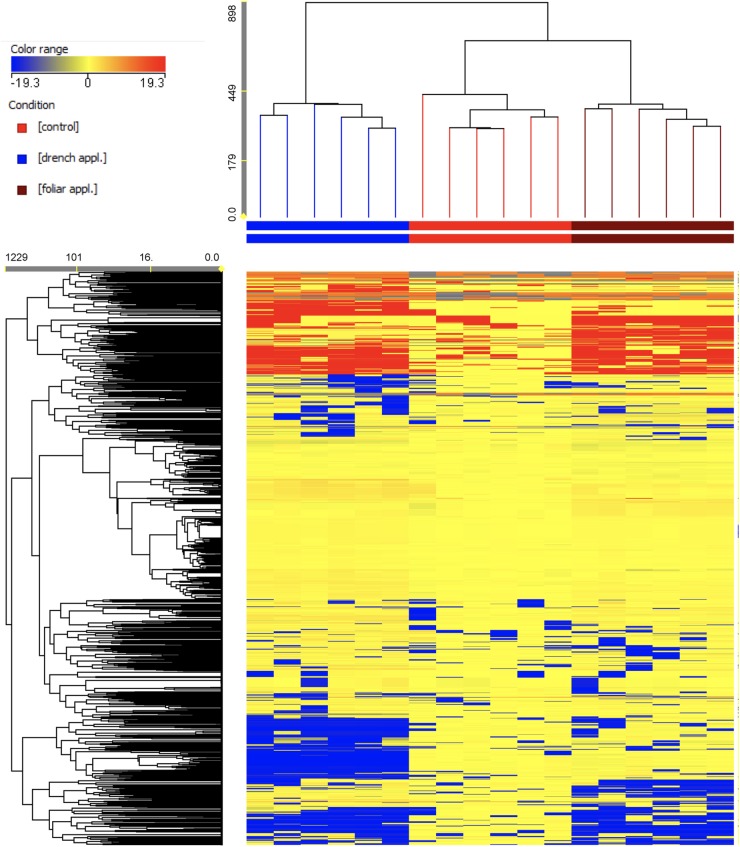
Unsupervised hierarchical cluster analysis (Euclidean similarity; linkage rule: Ward’s) carried out from metabolite profiles in tomato leaves from protein hydrolysate treated or untreated plants, as gained from UHPLC liquid chromatograph coupled to a quadrupole-time-of-flight mass spectrometer (UHPLC/QTOF-MS) untargeted metabolomics. Compound intensity was used to produce fold-change-based heat maps, based on which clustering was done.

The first step of interpretation was a hierarchical clustering, produced from the fold-change-based heatmap according to Euclidean distances. This unsupervised clustering approach allowed describing similarities/dissimilarities among treatments, as shown in Figure 4. As provided, two main clusters were generated–one comprising drench application and the other including foliar application and control. In this latter cluster, two distinct subclusters could be identified, thus indicating different metabolic profiles between foliar application of the biostimulant and control plants. Even though the application of PHs resulted in distinctive profiles in tomato under limited water availability, the naive (unsupervised) hierarchical clustering of metabolomic signatures suggested that the application method of the PH was an additional and relevant factor determining the actual difference in such phytochemical profiles.

**FIGURE 5:**
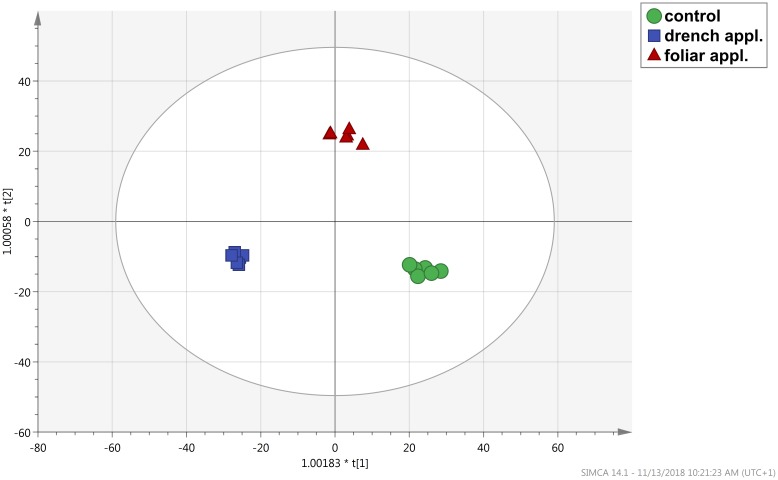
Score plot of Orthogonal Projection to Latent Structures Discriminant Analysis (OPLS-DA) supervised analysis carried out from metabolite profiles in tomato leaves from protein hydrolysate treated or untreated plants, as gained from UHPLC/QTOF-MS untargeted metabolomics.

A consistent outcome could be produced through the supervised OPLS-DA multivariate modeling. This analysis allowed separating predictive and orthogonal components (i.e., those components ascribable to technical and biological variation) of variance. Therefore, OPLS-DA effectively discriminated among the three groups into the score plot hyperspace. The OPLS-DA score plot (Figure 5) indicated a complete separation among control, foliar, and drench applications. The model parameters of the OPLS-DA regression were excellent, being *R*^2^*Y* = 0.99 and *Q*^2^*Y* = 0.94, respectively. The model was validated (CV-ANOVA *p* = 2.47 × 10^−10^) and overfitting could be excluded through permutation testing (*N* = 100). Validation through a misclassification table indicated a 100% model accuracy (Fisher’s probability 3.5 × 10^−7^). Furthermore, Hotelling’s T2 allowed us to exclude suspect and strong outliers. Given the validated model outcomes, the variable selection method called VIP (Variable Importance in Projection) was used to identify compounds explaining the differences observed. The discriminating compounds having a VIP score >1.25 were exported and subjected to fold-change analysis to identify the trends of regulation altered by the treatments. Thereafter, one-way ANOVA (Tukey *post hoc*) was used to describe significance of the differences. The discriminant compounds, together with their VIP score, *P*, and fold-change values, were grouped into chemical classes to facilitate the discussion of results (Table 1).

**Table 1:** Metabolites discriminating biostimulant-treated tomato plants (foliar and drench application) from control; results were gained from UHPLC/QTOF-MS untargeted metabolomics followed by OPLS-DA supervised statistics.

Compound		VIP score	VIP SE	*p*-Value	Log FC (foliar appl. vs. control)		Log FC (drench appl. vs. control)	
Lipids	A 1-acyl-sn-glycero-3-phosphoethanolamine (n-C14:1)	1.42	0.21	1.41E-24	−17.65	Down	−17.38	Down
	(5Z)-(15S)-11-alpha;-hydroxy-9,15-dioxoprostanoate	1.41	0.27	1.41E-24	−19.81	Down	−19.55	Down
	1-Palmitoyl-2-vernoloyl-phosphatidylcholine	1.39	0.20	2.48E-02	0.18	Up	−8.64	Down
	1-18:1-2-*Trans*-16:1-phosphatidylglycerol	1.39	0.44	2.07E-05	−1.38	Down	0.05	Up
	Dipalmitoyl phosphatidate	1.36	0.37	9.07E-05	0.18	Up	0.38	Up
	Phytosphingosine 1-phosphate	1.36	0.31	6.43E-23	−0.38	Down	−21.52	Down
	Arachidoyl dodecanoate	1.36	0.28	NS	−	−	0.20	Up
	14-Oxolanosterol/4-alpha-formyl,4-beta,14-alpha-dimethyl-9-beta,19-cyclo-5-alpha-cholest-24-en-3-beta-ol	1.35	0.31	1.19E-03	0.13	Up	−15.58	Down
	All-*trans*-heptaprenyl diphosphate	1.33	0.50	3.09E-21	0.34	Up	18.12	Up
	Sphinganine 1-phosphate	1.33	0.36	9.11E-22	−0.37	Down	−21.38	Down
	4-Alpha-formyl-stigmasta-7,24(24^1^)-dien-3-beta-ol	1.35	0.31	1.19E-03	0.13	Up	−15.58	Down
	Stearate	1.35	0.57	5.09E-03	13.73	Up	−2.40	Down
	9,10-Epoxy-18-hydroxystearate	1.35	0.55	NS	11.39	Up	10.34	Up
	(9Z)-12,13-Dihydroxyoctadeca-9-enoate	1.35	0.55	2.68E-02	11.39	Up	10.34	Up
	1-18:3-2-18:3-Monogalactosyldiacylglycerol	1.34	0.38	NS	−1.73	Down	−8.88	Down
	1-18:2-2-18:2-Monogalactosyldiacylglycerol	1.35	0.32	NS	−1.69	Down	−4.66	Down
	1-18:3-2-16:2-Monogalactosyldiacylglycerol	1.28	0.34	3.23E-02	−14.89	Down	−3.58	Down
	1-18:2-2-16:1-Phosphatidate	1.31	0.17	6.84E-05	−2.95	Down	−18.17	Down
	Vernoleate	1.38	0.33	4.67E-03	13.71	Up	12.14	Up
	(9R,10S)-Dihydroxystearate	1.34	0.15	NS	4.32	Up	−0.16	Down
	(9S,10S)-9,10-Dihydroxyoctadecanoate	1.34	0.15	NS	4.32	Up	−0.16	Down
	4-Hydroxybutanoate	1.37	0.32	1.01E-08	0.14	Up	3.28	Up
	9-*cis*-10′-apo-beta-carotenal	1.27	0.44	8.61E-04	−10.72	Down	−19.94	Down
	Farnesyl diphosphate	1.27	0.47	4.12E-05	0.63	Up	1.61	Up
	Epsilon, epsilon-carotene-3-diol/beta-carotene 15,15′ epoxide	1.31	0.42	1.57E-03	−17.52	Down	−17.62	Down
	All-*trans*-4,4′-diapolycopene	1.33	0.36	3.24E-12	0.05	Up	−7.17	Down
	Lutein	1.24	0.35	6.84E-05	3.42	Up	−15.52	Down
Resin acids	Palustradienal	1.51	0.37	0.00E+00	23.29	Up	4.07	Up
	Dehydroabietadiene	1.36	0.54	3.75E-04	1.31	Up	0.57	Up
	levopimaradiene/palustradiene/abieta-7,13-diene	1.39	0.35	1.57E-03	1.46	Up	0.22	Up
Triterpenes	Glycyrrhetinate/gypsogenin	1.39	0.22	3.24E-12	0.20	Up	−6.76	Down
	Betulinic aldehyde/ursolic aldehyde/11-oxo-beta-amyrin	1.35	0.31	1.19E-03	0.13	Up	−15.58	Down
Hormones	Gibberellin A98	1.36	0.24	9.07E-24	0.03	Up	−18.86	Down
	Indole-3-acetyl-phenylalanine	1.34	0.34	1.04E-21	−0.42	Down	−19.73	Down
	Indole-3-butyryl-glucose	1.34	0.35	3.97E-22	−0.28	Down	−20.55	Down
	A jasmonoyl-phenylalanine	1.33	0.32	1.59E-21	−0.42	Down	−20.51	Down
	Salicylate	1.29	0.57	NS	13.26	Up	18.76	Up
	Dihydrozeatin-7-N-glucose/dihydrozeatin-9-N-glucose	1.29	0.35	6.30E-05	−3.68	Down	−21.15	Down
	Isopentenyladenine-9-N-glucoside/isopentenyladenine-9-N-glucoside	1.29	0.37	6.30E-05	−3.45	Down	−19.71	Down
	Gibberellin A4/gibberellin A20	1.25	0.62	1.80E-03	0.77	Up	0.39	Up
	7-Oxateasterone	1.30	0.41	2.59E-21	−	−	−20.97	Down
	Cathasterone	1.25	0.66	8.05E-03	2.30	Up	−12.73	Down
Osmolytes	Alpha, alpha-trehalose	1.40	0.37	3.68E-02	14.23	Up	0.62	Up
	Glycine betaine	1.33	0.49	1.49E-02	−0.57	Down	−0.23	Down
Polyamines	Triferuloyl spermidine	1.28	0.18	NS	−2.29	Down	−9.75	Down
	Feruloylserotonin	1.34	0.35	1.96E-22	−0.15	Down	−19.56	Down
	Serotonin	1.29	0.41	3.35E-20	−0.30	Down	−18.65	Down
	p-Coumaroyltyramine	1.31	0.46	0.001	3.51	Up	−11.96	Down
	Sinapoyltyramine	1.34	0.18	0.001	18.77	Up	0.60	Up
Pteridins	2-Amino-6-carboxamido-7,8-dihydropteridin-4-one	1.31	0.47	1.97E-02	9.34	Up	10.70	Up
	5,10-Methylenetetrahydropteroyl mono-L-glutamate	1.25	0.25	6.51E-04	−6.33	Down	−18.05	Down
	10-Methyl-5,6,7,8-tetrahydropteroylglutamate	1.37	0.41	1.91E-22	−17.33	Down	−17.06	Down
Chlorophyll	Red chlorophyll catabolite	1.33	0.28	NS	6.73	Up	20.70	Up
	Coproporphyrinogen III	1.32	0.40	0.001	−0.66	Down	−0.87	Down
	Coproporphyrin III	1.34	0.42	0.001	−0.84	Down	−0.54	Down
	Pyropheophorbide *a*	1.31	0.32	NS	0.35	Up	0.83	Up
	Coproporphyrin I	1.26	0.72	0.001	−1.11	Down	−0.99	Down
Quinones	Phylloquinone	1.31	0.37	NS	−	−	−5.22	Down
	Demethylphylloquinol	1.35	0.31	1.19E-03	0.13	Up	−15.58	Down
	2-Heptyl-3-hydroxy-4(1H)-quinolone	1.35	0.41	NS	16.38	Up	22.27	Up
	3″-Hydroxy-geranylhydroquinone	1.34	0.66	1.17E-04	15.86	Up	0.60	Up
Others	(S)-Coclaurine	1.43	0.41	6.24E-05	2.20	Up	1.11	Up
	Coumarinic acid-beta-D-glucoside	1.46	0.17	3.36E-22	−19.86	Down	−0.78	Down
	3-Methoxy-4-hydroxy-5-hexaprenylbenzoate	1.40	0.16	7.52E-12	0.17	Up	−6.09	Down
	A 6-hydroxy-5-isopropenyl-2-methylhexanoate	1.39	0.25	6.70E-05	8.10	Up	7.56	Up
	Casbene	1.39	0.35	1.57E-03	1.46	Up	0.22	Up
	N,N-dihydroxy-L-isoleucine	1.36	0.21	6.93E-10	−0.17	Down	−2.53	Down
	Secologanin	1.36	0.35	8.83E-04	−0.99	Down	−0.38	Down
	Adenosine pentaphosphate	1.35	0.28	0.00E+00	16.57	Up	16.17	Up
	3-Hydroxy-16-methoxy-2,3-dihydrotabersonine	1.34	0.33	3.57E-22	−0.45	Down	−22.12	Down
	Thymidine	1.34	0.52	1.30E-19	−17.85	Down	−17.59	Down
	L-Valine	1.33	0.49	NS	−0.57	Down	−0.23	Down

*Compounds are grouped into biochemical classes and are presented with their individual VIP score and standard error (SE), as well as p-Value (one-way ANOVA, Bonferroni multiple testing correction) and Log of fold-change values. NS, not significant (p > 0.05). Missing values denote fold-change values < 1.5. VIP, Variable Importance in Projection; UHPLC/QTOF-MS, UHPLC liquid chromatograph coupled to a quadrupole-time-of-flight mass spectrometer; OPLS-DA, Orthogonal Projections to Latent Structures Discriminant Analysis.*

Notably, relatively few biochemical classes included most of the discriminant metabolites. In more detail, lipids (including membrane lipids, sterols, carotenoids, and other terpenes) were the most represented class of compounds among VIP discriminants, followed by phytohormones, polyamine conjugates, prenyl quinones, and chlorophyll-related compounds. Among hormones, brassinosteroids, indole conjugates, salicylate, cytokinins, and two gibberellins were identified among discriminant compounds of treatments (Table 1). Furthermore, abietane diterpene resin acids, as well as pteridins and few other compounds, could be outlined by VIP analysis. Interestingly, two osmolytes (trehalose and glycine betaine) were identified among VIP discriminants (Table 1).

The following chemical enrichment analysis carried out in chemRICH highlighted sterols (cholestanes, cholestadienols, and hydroxycholesterols), carotenoids, unsaturated fatty acids and phosphatidic acids, terpenes, and coproporphyrins as the most represented chemical groups (Supplementary Figure S6). The analysis, carried out separately for each application method (foliar or drench as compared to control), represented differences in accumulation for the selected metabolites. Most of the classes reported exhibited a down-accumulation following biostimulants treatment, as compared to control, except for terpenes (foliar application treatment) and unsaturated fatty acids (drench application treatment).

## Discussion

The biostimulant effect on sink and source organs is clearly visible in this study. PH biostimulant directly enters sink areas like the roots through drenching application, while the same biostimulant, foliarly sprayed, directly enters the source region, the shoot and leaves. This may be reflected in photosynthetic and physiologic functions differently. Regulation of stomatal function is an important mechanism in dealing with the adverse consequences of limited water availability. The typical response of plants to water limitation is stomatal closure, through which the amount of water loss through transpiration can be decreased. On the other hand, water stress-induced closing of stomata also limits CO_2_ uptake; therefore, it decreases the efficiency of net photosynthesis. Drenched PH application affected the physiological and metabolic activity of plants. This could be due to enhanced stomatal conductance activity of drench application of PH through the sink region. Russell et al. (2006) reported that other biostimulant substances like humic fractions promoted stomatal opening in pea with a broad biphasic concentration dependence. The extent of opening was similar to that produced by auxin, and a component sensitive to inhibitors of calcium-independent phospholipase A2 was involved in signaling the response to humic fractions and auxin (Russell et al., 2006). Moreover, tomato plants drenched with PH obtained a more favorable balance between carbon gain and water loss as shown by the increase of TUE. The reduced CO_2_ uptake imposed by limited water availability causes an imbalance between PSII activity and the following carbon assimilation via the Calvin cycle, thus increasing the excitation energy on PSII and inducing photodamage (Baker and Rosenqvist, 2004).

Furthermore, it is known that the water-related osmotic stress generates a secondary oxidative stress. Reactive oxygen species (ROS) are produced via incomplete reduction of oxygen (O_2_^•−^) and are known as signaling molecules integrated with hormone signaling networks (Foyer, 2018). The specific application mode for the PH biostimulant imposed a wide variation of phytohormone profile. Two brassinosteroids (teasterone and cathasterone), a class of sterol-like hormones linked to several signaling networks including abiotic stress response, cell wall development, and lignification, were detected. In more detail, brassinosteroids are reported to be involved in water stress resistance and osmotic stress-induced stomatal closure as well as to mediate ROS formation, jasmonate signaling, and abscisic acid (ABA) response (Lee et al., 2018; Lucini et al., 2018). ABA and cytokinins antagonistically regulate environmental stress responses in plants, and their integrated and coordinated action modulates drought stress response (Huang et al., 2018). Indeed, cytokinins were down-accumulated, following both foliar and drench application. In plants, cytokinin signaling involves a canonical two-component system that comprises histidine kinases and histidine phosphotransfer proteins. Considering that cytokinin signaling components have been shown to act as negative regulators of plant tolerance to limited water availability (Huang et al., 2018), the trend observed following biostimulant application might represent a significant contribution in water stress resistance. Salicylic acid is another phytohormone that plays a pivotal role in mediating water stress response via modulation of ROS production and redox state (La et al., 2019). Salicylic acid, together with jasmonate, has also been found to enhance water stress tolerance in plants (Li et al., 2018). The application of the PH biostimulant imposed a marked up-accumulation of salicylate, thus potentially modulating with ROS accumulation, ROS-mediated signaling, and tolerance to low water availability. Indeed, salicylate mediates redox balance with an antagonistic depression of ABA (La et al., 2019). Auxins are well-known phytohormones that promote root initiation and delay plant senescence (Li et al., 2018); interestingly, two conjugated forms (i.e., storage forms) of indoleacetic acid (IAA) were found down-accumulated following both PH treatments. The PH-mediated hydrolysis of IAA conjugates may have generated free IAA, leading to stimulation of stomatal opening in PH-treated plants. Besides affecting hormone profile, limited water availability conditions impair the consumption of reduction equivalents for CO_2_ fixation, thus resulting in an oversupply of NADPH. Therefore, metabolic processes are expected to push toward the synthesis of highly reduced compounds (Radwan et al., 2017). With this regard, the increase in farnesyl diphosphate and triterpenes is not surprising. Consistently, Nasrollahi et al. (2014) reported a drought-induced accumulation of triterpenes.

Several other lipids, including membrane lipids and carotenoids, were modulated by biostimulant application under limited water availability conditions. Although a clear trend could not be outlined, membrane lipids are known to be altered under plant stress conditions and to play a role in plant adaptation to stress (Allakhverdiev et al., 2001; Lucini et al., 2015; Rouphael et al., 2016). These membrane components are involved in the production of signaling molecules, and they are regulated by plant signaling under abiotic stress (Hou et al., 2016). Indeed, lipid-dependent signaling cascades contribute to trigger plant adaptation processes (Hou et al., 2016).

In the current study, hydroxycinnamic amides (two tyramine derivatives, a serotonin, and a spermidine conjugate) were also induced by biostimulant application. This accumulation was observed for tyramine conjugates. It is interesting to note that biogenic amines and their hydroxycinnamic amides act in plants by interacting with phytohormone cross-talk together with mediating root growth and ROS signaling (Mukherjee, 2018). In particular, tyramine hydroxycinnamic amides are said to also stimulate wound healing and suberization processes (Voynikov et al., 2016). Nonetheless, exogenous polyamines are reported to alleviate the drought-induced detrimental effects as well as to alter auxins, zeatin, gibberellins, salicylic acid, and jasmonate (Li et al., 2018). Abietane diterpene resin acids were also stimulated by the treatment, particularly concerning palustric acid intermediates. These diterpenes are reported to function as antioxidants to protect membranes from oxidative stress (Munné-Bosch et al., 1999) and to display antibacterial and antifungal activity (Helfenstein et al., 2017).

An osmolyte, namely, the trehalose, was found to be up-accumulated following biostimulant treatment under water scarcity. Indeed, the accumulation of sugars, predominantly trehalose, is a known protection mechanism in plants experiencing abiotic stresses, since they contrast protein denaturation, scavenge free radicals, and stabilize biological membranes (Asaf et al., 2017; Farooq et al., 2018). Trehalose, in particular, is able to bind to the polar region of membranes to scavenge the ROS (Farooq et al., 2018).

The involvement of prenyl quinones, generally found up-accumulated, suggests the enrollment of both signaling and antioxidant functions under oxidative stress. The chloroplastic pool of these compounds is related to the oxidation by the cytochrome *b6f* complex as well as to other thylakoid electron transfer pathways. The modulation of such prenyl quinones has been related to their function as signaling molecules in chloroplast-to-nucleus signal transduction and is involved in plant acclimation to stress (Kruk et al., 2016). Finally, among others, intermediates (tetrapyrrole coproporphyrins) and catabolites (pheophorbide *a*) of chlorophyll biosynthetic pathway(s) were identified among VIP discriminants. The former were down-accumulated in treated plants, whereas an opposite trend could be observed for pheophorbide *a*. Ghandchi et al. (2016) reported that the degradation of chlorophyll to non-fluorescent pigments is a transcriptionally regulated intricate process that varies during the plant life cycle. These authors also suggested that the activity of the degrading enzyme pheophorbide *a* oxygenase (PAO) is altered by drought. Nonetheless, it is important to consider that chlorophyll intermediates play a pivotal role also in ROS signaling and production. Photoreduction of oxygen to the superoxide radical is related to a reduced electron transport in PSI and to a reaction linked to the photorespiratory cycle occurring in the peroxisome. This second process is enhanced under drought because of the limited availability of CO_2_. Unlike mammals (where ROS are mainly produced in mitochondria), plants produce singlet oxygen mainly in thylakoids by chlorophyll and its tetrapyrrole intermediates in the presence of light. These compounds are partially hydrophobic and are therefore associated with the thylakoid membranes, which do not form pigment protein complexes. Considering that most carotenoids are located in the pigment–protein complexes, they are spatially far from tetrapyrroles and therefore they are poorly effective in quenching their triplet states (Tripathy and Oelmüller, 2012). Therefore, coproporphyrins act as photosensitizers and their accumulation leads to light-dependent necrosis in plant (Hu et al., 1998; Ishikawa et al., 2001). On this basis, it can be postulated that the biostimulant-related down-accumulation of coproporphyrins under limited water availability can represent a key factor to mitigate ROS imbalance and to improve drought tolerance. Moreover, photosynthetic organisms can dissipate excess energy *via* non-photochemical quenching to avoid singlet oxygen formation; carotenoids play a crucial role in such non-photochemical quenching (Tripathy and Oelmüller, 2012). These findings suggest a complex and coordinated regulation of ROS under limited water availability involving both isoprenoid quinones and tetrapyrrole intermediates. Consistently, several carotenoids, as well as their epoxy- and diol-derivatives, were down-accumulated in biostimulant-treated tomato plants. These findings support and strengthen our previous evidence related to an improved capability of PH-treated tomato plants to cope with ROS-mediated oxidative stress.

Nonetheless, such biochemical reprogramming can be linked to the specific characteristics of PH biostimulants. In fact, it has been reported that peptides in PHs can activate signaling cascades in plant, including the elicitation of defense mechanisms against oxidative stress (Ertani et al., 2009; Percival, 2010; Storer et al., 2016; Lucini et al., 2018). Such cascade of events is typically hormone-mediated (Lucini et al., 2015, 2016, 2018). Some other components of PHs, such as free amino acids, might support the biostimulant activity we observed. A direct provision of glycine and proline might promote osmolyte accumulation, whereas tryptophan is a biosynthetic precursor of indoles and auxins in particular. The direct provision of antioxidant compounds could also be postulated, given the content of phenolics and peptides in the test product. Therefore, a coordinate action of different compounds might have induced the molecular alterations we observed via metabolomics. On the other hand, such classes of biologically active compounds are available to plants following application of PHs. Peptides could enter the leaves through the stoma following foliar application, rather than via ABC membrane transporters following drench application (Boursiac et al., 2013). However, smaller compounds can also use hydrophilic pores in leaves and other transporters in root. In fact, evidence indicated that hydrophilic solutes penetrate cuticles via a physically distinct pathway other than simple diffusion in the cuticle, and they are called “polar pores” (Fernandez and Eichert, 2009).

Therefore, although further investigation is advisable to better elucidate the complex mechanisms of interaction between biostimulants and plant, the modulation of the molecular signatures we observed can be connected to PH application.

## Conclusion

Our findings indicate that PH application on tomato plants can be considered as a sustainable crop enhancement technology for agricultural productivity under water-limited conditions. Mining of variations in growth dynamics and physiological responses was clearly qualitatively and quantitatively phenotyped using high-throughput phenomic tools. Morpho-physiological data suggest that PH application, especially using the substrate drench method, can be recommended as a highly sustainable approach under less water available conditions. PH application in drenching mode causes plants to transpire more and increase stomatal conductance leading to a better TUE; however, light absorption parameters were unaffected by inducing higher redox status. The UHPLC-QTOF-MS metabolomic approach allowed the identification of the molecular bases of the improved water stress tolerance following biostimulant treatment. Our approach identified a distinct metabolic signature imposed by drench or foliar application of the PH under limited water availability in tomato, as highlighted by both unsupervised hierarchical clustering and supervised discriminant analysis. These outcomes supported and integrated phenomic outcomes, indicating the biochemical processes implicated in the enhanced tolerance to limited water availability following biostimulant application. In more detail, a wide and organized range of metabolic processes was involved in response of tomato plants to PH treatments. Phytohormone profile was significantly affected, even though the most represented among differential compounds were lipids (including membrane lipids, sterols, and terpenes). As a general overview, PH-treated tomato plants exhibited an improved tolerance to ROS-mediated oxidative imbalance. Such tolerance involved a coordinated action of salicylic acid, hydroxycinnamic amide signaling, carotenoids, and prenyl quinone radical scavenging, as well as reduced tetrapyrrole biosynthesis. Finally, further studies are advisable to understand if the biostimulant activity observed with foliar and drench applications of PH is related to changes of microbial community at the leaf or root level.

## Author Contributions

KeP wrote the first draft of the manuscript, followed the phenotyping measurements, and contributed to phenotype data interpretation. MS performed the big data analysis. LL, MM, and PB performed the metabolomics analysis, data interpretation, and wrote the metabolomic part. KlP, YR, MC, HR, RC, MT, and GC were involved in data analysis, data interpretation, and writing the manuscript. GC and KlP coordinated the whole project, provided the intellectual input, set up the experiments, and corrected the manuscript.

## Conflict of Interest Statement

MT is the owner and CEO of PSI (Photon Systems Instruments), Drásov, Czechia, and KlP is an employee of his company. KeP is an ex-employee of PSI, and an MS and a Ph.D. student both conducted the experiments at PSI. RC is the director of Nixe Company. HR is an employee of Italpollina Company (Anderson, CA, United States). GC is a member of the spin-off company Arcadia approved by Tuscia University, Italy. The remaining authors declare that the research was conducted in the absence of any commercial or financial relationships that could be construed as a potential conflict of interest.

## Footnotes

1 http://www.plantcyc.org; released April 2018

2 http://cosmos-fp7.eu/msi.html

3 https://pubchem.ncbi.nlm.nih.gov/

4 http://chemrich.fiehnlab.ucdavis.edu

## Supplementary Material

FIGURE S1 Schematic overview of plant handling and phenotyping protocol. **(A)** Plant phenotyping was carried out in the PlantScreen^TM^ Modular System installed in semi-controlled greenhouse environment conditions in the PSI Research Center. Tomato plants were transferred from a controlled environment to the phenotyping system and automated phenotyping protocol was initiated. Plants were regularly screened using a kinetic chlorophyll fluorescence imaging unit, a calibrated RGB camera for top and multiple-angle side projections, and a thermal imaging unit. A low irrigation level watering regime was maintained by regular weighing and watering (WW) of the plants by an automated WW unit. **(B)** Protein hydrolysate biostimulant application protocol. Tomato plants were treated with PHs either by spraying (foliar application) or by drenching (drench application). Following the PHs application, plants were transferred back to the control environment and were kept under high-humidity conditions for the following 24 h.

FIGURE S2 Destructive biomass quantification and correlation with digital biomass. **(A)** Fresh and dry weight of tomato shoots harvested following the end of the phenotyping period (day 19). Values represent the average of six biological replicates per treatment. Error bars represent standard deviation. Different letters indicate significant difference according to one-way ANOVA *post hoc* Tukey’s test (*p* < 0.05). **(B)** Correlation of digital shoot biomass (px) acquired on day 15 with fresh weight (g) of tomato plants harvested on day 19 of phenotyping period. **(C)** Correlation of digital shoot biomass (px) acquired on day 15 of phenotyping period with dry weight (g) of tomato plants harvested at the end of phenotyping period.

FIGURE S3 Variation in shoot colors of tomato plants prior to and following the biostimulant treatment. Dynamic relative changes in greenness hue abundance over the phenotyping period in control tomato plants and plants treated with PH either by spraying or drenching. The six most representative color hues are shown in RGB color scale as percentage of the shoot area (pixel counts) of six biological replicates per treatment.

FIGURE S4 Photosynthetic performance of the tomato plants. The photochemical quenching coefficient that estimates the fraction of closed PSII reaction centers (1 - *q_P_*), steady-state non-photochemical quenching (NPQ), and electron transport rate (ETR) was measured using the light curve protocol. Data are mean of six independent plants per treatment. Measurements at three actinic photon irradiance intensities were acquired. Measurements were taken at 170, 620, and 1070 μmol photons m^−2^ s^−1^, respectively.

FIGURE S5 Leaf temperature quantification and estimation of transpiration use efficiency (TUE) in tomato plants prior to and following PH treatment. **(A)** Leaf temperature was quantified by thermal imaging. To minimize the influence of the environmental variability and the difference in the image acquisition timing among individual plants, raw temperature of each plant (°C) was normalized by the actual background temperature. Temperature of leaves of the plants was determined as the difference relative to the surrounding air temperature and was expressed as Δ*T* (°C). Air temperature data were obtained from a reference surface, which is in thermal equilibrium with air in the background of the plant. **(B)** TUE was estimated from transpiration and growth, measured by water loss and pixel counts over the whole experimental period, respectively. Values represent the average of six biological replicates per treatment. Error bars represent standard deviation. Different letters indicate significant difference according to one-way ANOVA *post hoc* Tukey’s test (*p* < 0.05).

FIGURE S6 Chemical Similarity Enrichment Analysis (ChemRICH) carried out from discriminant metabolites in biostimulant-treated tomato plants. Enrichment analysis is based on chemical similarities and uses Tanimoto substructure chemical similarity coefficients to cluster metabolites into non-overlapping chemical groups. Distinct analyses were performed for foliar **(A)** and drench application **(B)**.

TABLE S1 Projected shoot area (PSA) of the tomato plants cultivated under limited irrigation and subjected to treatment by PH either by spraying or drenching. PSA values were extracted from multiple side view RGB images and are expressed as number of green pixels and represent the average of six biological replicates per treatment ± standard deviation. Within the same row and for the specified day different letters indicate significant difference according to one-way ANOVA *post hoc* Tukey’s test (*p* < 0.05).

TABLE S2 Projected shoot area (PSA) of the tomato plants cultivated under limited irrigation and subjected to treatment by PH either by spraying or drenching. PSA values were extracted from top view RGB images and are expressed as number of green pixels and represent the average of six biological replicates per treatment ± standard deviation. Within the same row and for the specified day different letters indicate significant difference according to one-way ANOVA *post hoc* Tukey’s test (*p* < 0.05).

TABLE S3 Digital biomass of tomato plants cultivated under limited irrigation and subjected to treatment by PH either by spraying or drenching. Values are expressed as number of green pixels and represent the average of six biological replicates per treatment ± standard deviation. Within the same row and for the specified day different letters indicate significant difference in digital biomass, according to one-way ANOVA *post hoc* Tukey’s test (*p* < 0.05).

TABLE S4 Width of the tomato plants extracted from multiple side view RGB images of the tomato plants cultivated under limited irrigation and subjected to treatment by PH either by spraying or drenching. Values are expressed as number of green pixels and represent the average of six biological replicates per treatment ± standard deviation. Within the same row and for the specified day different letters indicate significant difference according to one-way ANOVA *post hoc* Tukey’s test (*p* < 0.05).

TABLE S5 Height of the tomato plants extracted from multiple side view RGB images of the tomato plants cultivated under limited irrigation and subjected to treatment by PH either by spraying or drenching. Values are expressed as number of green pixels and represent the average of six biological replicates per treatment ± standard deviation. Within the same row and for the specified day different letters indicate significant difference according to one-way ANOVA *post hoc* Tukey’s test (*p* < 0.05).

TABLE S6 Variation in shoot colors of tomato plants cultivated under limited irrigation and subjected to treatment by PHs either by spraying or drenching. The values for 6 most representative color hues are shown as percentage of the shoot area (pixel counts). Values represent the average of six biological replicates per treatment ± standard deviation. Within the same row and for the specified day different letters indicate significant difference according to one-way ANOVA *post hoc* Tukey’s test (*p* < 0.05).

TABLE S7 Photosynthetic performance of tomato plants. Photosynthetic parameters deduced from kinetic chlorophyll fluorescence imaging on whole plant level in tomato plants cultivated under limited irrigation and subjected to treatment by PH either by spraying or drenching. Minimal fluorescence in dark-adapted state (F_0_ ), maximum fluorescence in dark-adapted state (F_M_ ), maximum quantum yield of PSII photochemistry for the light-adapted state (*F_v_*/*F*_m_), the photochemical quenching coefficient that estimates the fraction of open PSII reaction centers (qP), proportion of closed PSII reaction centers (1-qP), steady-state non-photochemical quenching (NPQ) and electron transport rate (ETR) were measured using the light curve protocol for tomato plants prior and upon two times of PHs treatments. Values represent the average of six biological replicates per treatment ± standard deviation. Within the same row and for the specified day different letters indicate significant difference according to one-way ANOVA *post hoc* Tukey’s test (*p* < 0.05). Lss1, Lss2, and Lss3 represent actinic photon irradiance measurements taken at 170, 620, and 1070 μmol photons m^−2^ s^−1^ PAR values, respectively.
