# Supplementary figures and images for: A Combined Phenotypic and Metabolomic Approach for Elucidating the Biostimulant Action of a Plant-Derived Protein Hydrolysate on Tomato Grown Under Limited Water Availability
Source: Front Plant Sci. 2019 May 3;10:493. doi: 10.3389/fpls.2019.00493 (PMC6509618; doi:10.3389/fpls.2019.00493)

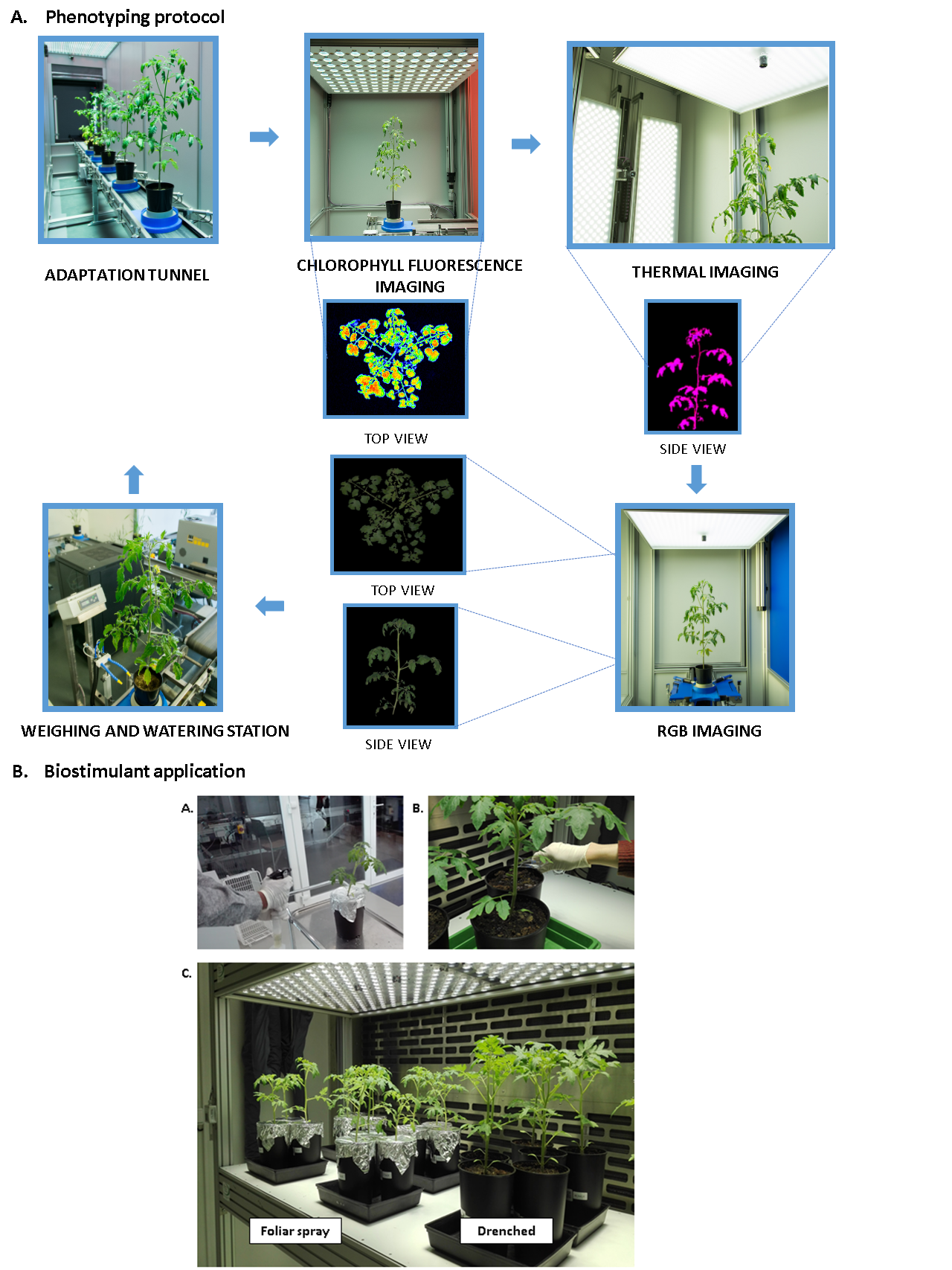

Supplement: FIGURE S1 — Schematic overview of plant handling and phenotyping protocol. (A) Plant phenotyping was carried out in the PlantScreenTM Modular System installed in semi-controlled greenhouse environment conditions in the PSI Research Center. Tomato plants were transferred from a controlled environment to the phenotyping system and automated phenotyping protocol was initiated. Plants were regularly screened using a kinetic chlorophyll fluorescence imaging unit, a calibrated RGB camera for top and multiple-angle side projections, and a thermal imaging unit. A low irrigation level watering regime was maintained by regular weighing and watering (WW) of the plants by an automated WW unit. (B) Protein hydrolysate biostimulant application protocol. Tomato plants were treated with PHs either by spraying (foliar application) or by drenching (drench application). Following the PHs application, plants were transferred back to the control environment and were kept under high-humidity conditions for the following 24 h. [file Image_1.PNG]

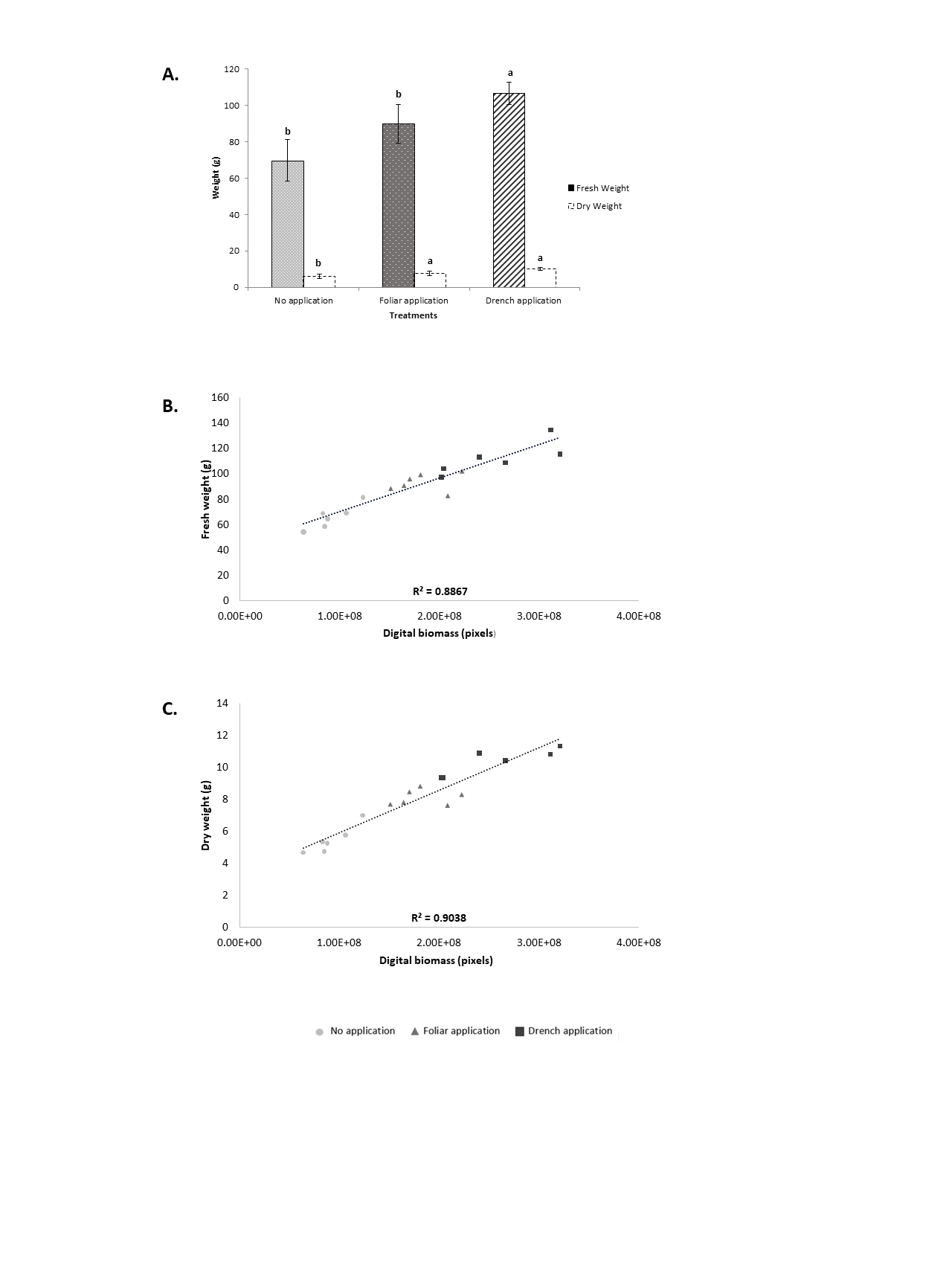

Supplement: FIGURE S2 — Destructive biomass quantification and correlation with digital biomass. (A) Fresh and dry weight of tomato shoots harvested following the end of the phenotyping period (day 19). Values represent the average of six biological replicates per treatment. Error bars represent standard deviation. Different letters indicate significant difference according to one-way ANOVA post hoc Tukey’s test (p < 0.05). (B) Correlation of digital shoot biomass (px) acquired on day 15 with fresh weight (g) of tomato plants harvested on day 19 of phenotyping period. (C) Correlation of digital shoot biomass (px) acquired on day 15 of phenotyping period with dry weight (g) of tomato plants harvested at the end of phenotyping period. [file Image_2.PNG]

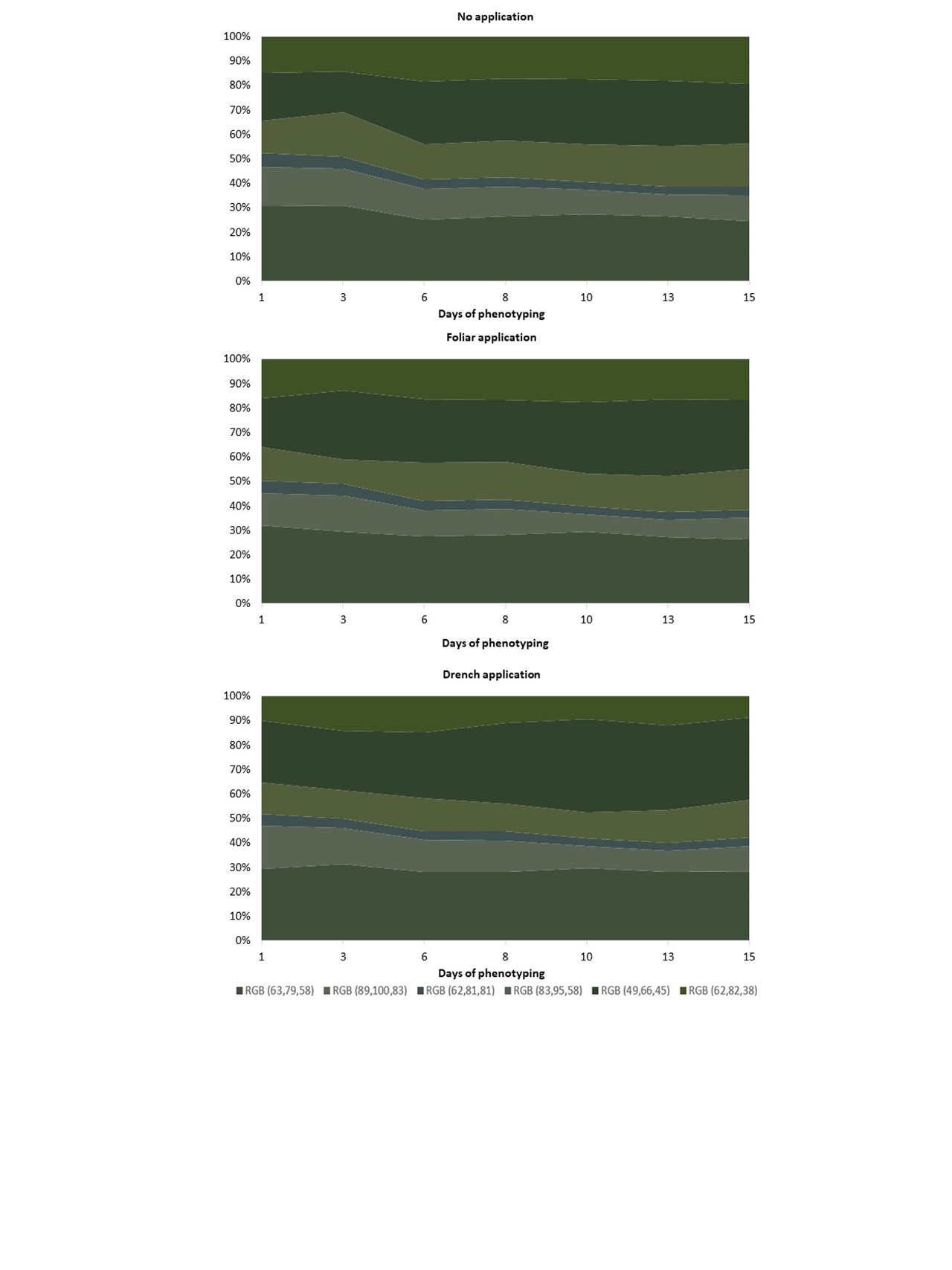

Supplement: FIGURE S3 — Variation in shoot colors of tomato plants prior to and following the biostimulant treatment. Dynamic relative changes in greenness hue abundance over the phenotyping period in control tomato plants and plants treated with PH either by spraying or drenching. The six most representative color hues are shown in RGB color scale as percentage of the shoot area (pixel counts) of six biological replicates per treatment. [file Image_3.PNG]

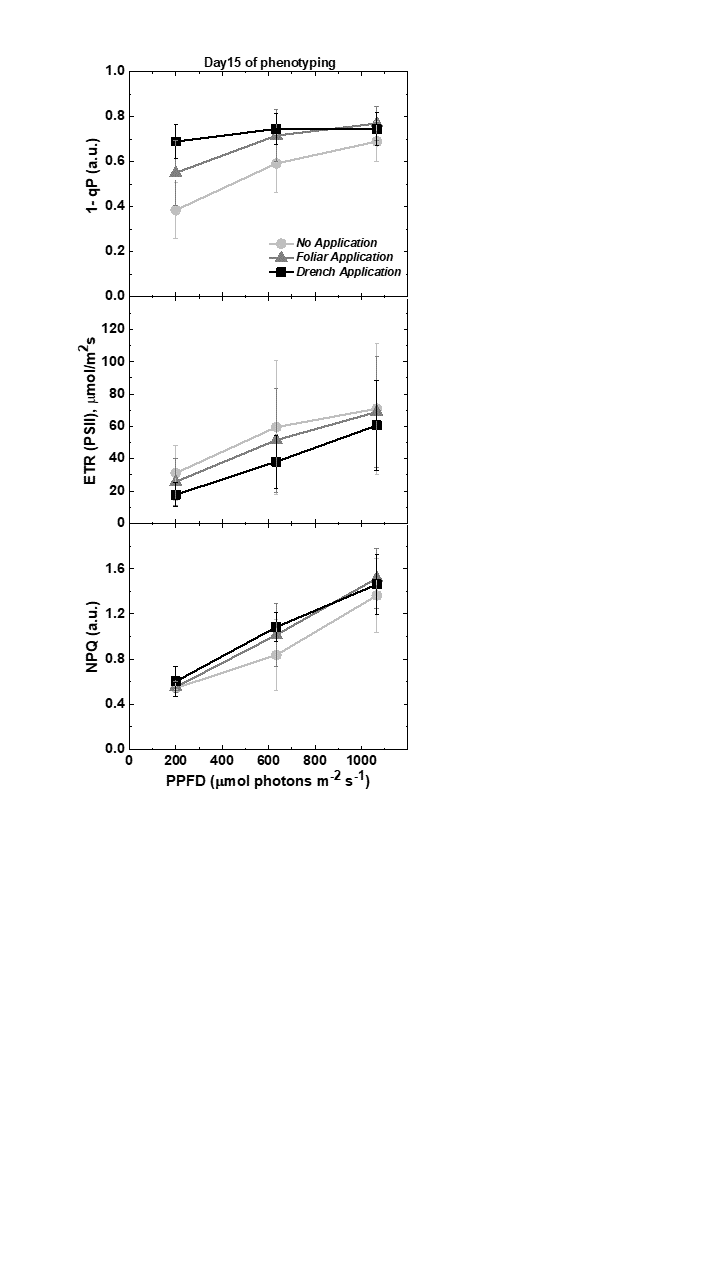

Supplement: FIGURE S4 — Photosynthetic performance of the tomato plants. The photochemical quenching coefficient that estimates the fraction of closed PSII reaction centers (1 - qP), steady-state non-photochemical quenching (NPQ), and electron transport rate (ETR) was measured using the light curve protocol. Data are mean of six independent plants per treatment. Measurements at three actinic photon irradiance intensities were acquired. Measurements were taken at 170, 620, and 1070 μmol photons m−2 s−1, respectively. [file Image_4.PNG]

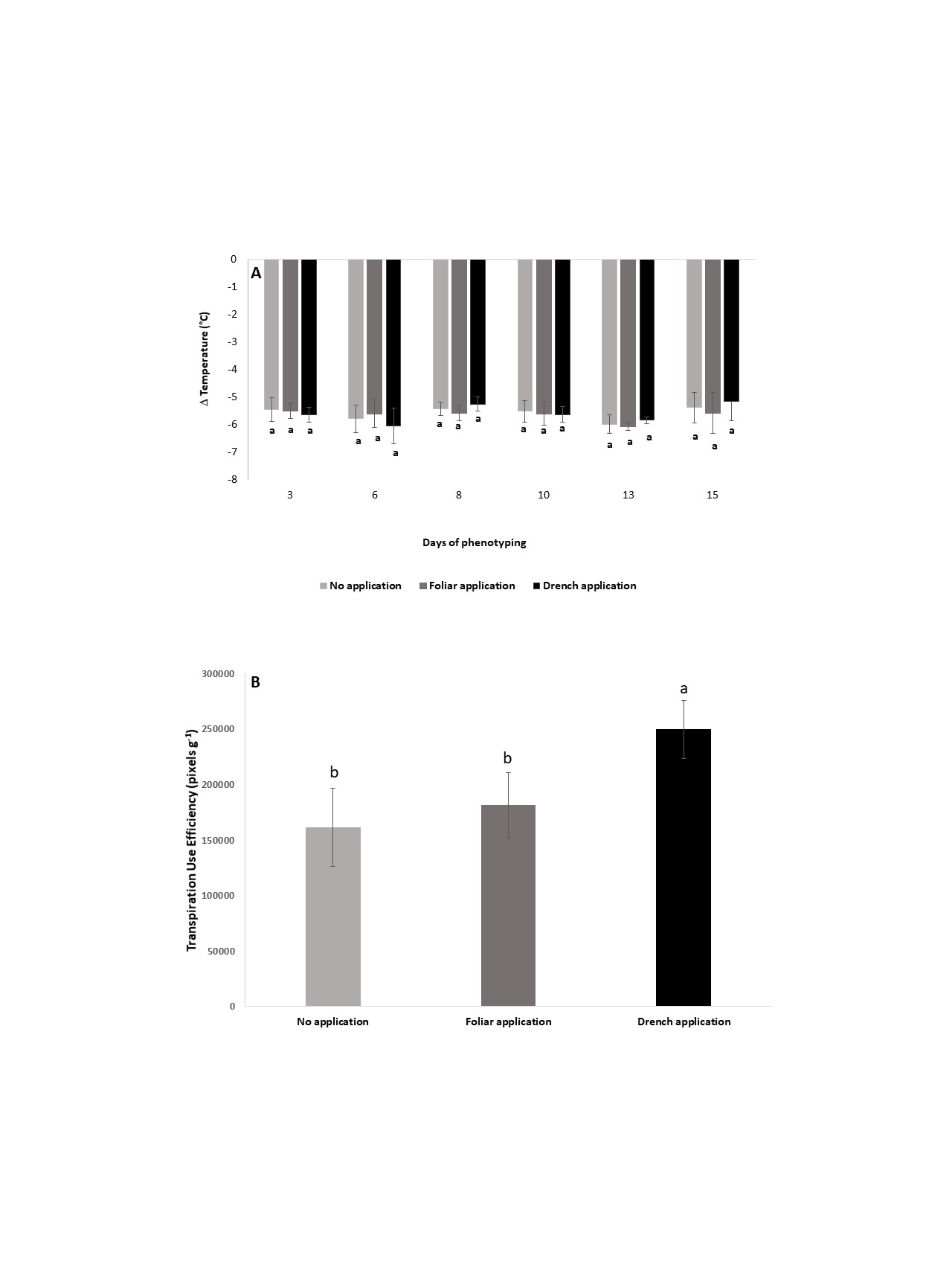

Supplement: FIGURE S5 — Leaf temperature quantification and estimation of transpiration use efficiency (TUE) in tomato plants prior to and following PH treatment. (A) Leaf temperature was quantified by thermal imaging. To minimize the influence of the environmental variability and the difference in the image acquisition timing among individual plants, raw temperature of each plant (°C) was normalized by the actual background temperature. Temperature of leaves of the plants was determined as the difference relative to the surrounding air temperature and was expressed as ΔT (°C). Air temperature data were obtained from a reference surface, which is in thermal equilibrium with air in the background of the plant. (B) TUE was estimated from transpiration and growth, measured by water loss and pixel counts over the whole experimental period, respectively. Values represent the average of six biological replicates per treatment. Error bars represent standard deviation. Different letters indicate significant difference according to one-way ANOVA post hoc Tukey’s test (p < 0.05). [file Image_5.JPEG]

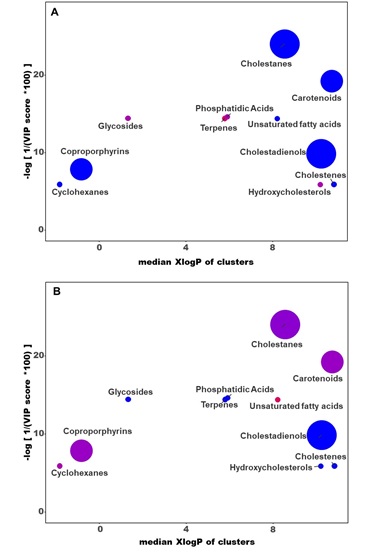

Supplement: FIGURE S6 — Chemical Similarity Enrichment Analysis (ChemRICH) carried out from discriminant metabolites in biostimulant-treated tomato plants. Enrichment analysis is based on chemical similarities and uses Tanimoto substructure chemical similarity coefficients to cluster metabolites into non-overlapping chemical groups. Distinct analyses were performed for foliar (A) and drench application (B). [file Image_6.JPEG]
