# Supplementary material for: A Combined Phenotypic and Metabolomic Approach for Elucidating the Biostimulant Action of a Plant-Derived Protein Hydrolysate on Tomato Grown Under Limited Water Availability
Source: Front Plant Sci. 2019 May 3;10:493. doi: 10.3389/fpls.2019.00493 (PMC6509618; doi:10.3389/fpls.2019.00493)
Supplement: TABLE S1 — Projected shoot area (PSA) of the tomato plants cultivated under limited irrigation and subjected to treatment by PH either by spraying or drenching. PSA values were extracted from multiple side view RGB images and are expressed as number of green pixels and represent the average of six biological replicates per treatment ± standard deviation. Within the same row and for the specified day different letters indicate significant difference according to one-way ANOVA post hoc Tukey’s test (p < 0.05). [file Table_1.DOCX]

**Suppl. Table 1.** Projected shoot area (PSA) of the tomato plants cultivated under limited irrigation and subjected to treatment by PH either by spraying or drenching. PSA values were extracted from multiple side view RGB images and are expressed as number of green pixels and represent the average of six biological replicates per treatment ± standard deviation. Within the same row and for the specified day different letters indicate significant difference according to one-way ANOVA post-hoc Tukey’s test (p<0.05).

| Treatment | Day 6 | | Day 8 | | Day 10 | | Day 13 | | Day 15 | |
| --- | --- | --- | --- | --- | --- | --- | --- | --- | --- | --- |
| No application | 52746 ±  12318 | *bc* | 71389 ±  13402 | *cd* | 94289 ±  19056 | *c* | 158410 ±  35668 | *d* | 189604 ±  45074 | *e* |
| Foliar application | 58773 ±  13114 | *abc* | 82719 ±  15767 | *bc* | 114162 ±  21305 | *bc* | 186223 ±  35127 | *cd* | 237218 ±  41439 | *cd* |
| Drench application | 68336 ±  13632 | *a* | 90970 ±  10739 | *b* | 130816 ±  16357 | *b* | 224381 ±  33242 | *b* | 286059 ±  36138 | *b* |

**Suppl. Table 2.** Projected shoot area (PSA) of the tomato plants cultivated under limited irrigation and subjected to treatment by PH either by spraying or drenching. PSA values were extracted from top view RGB images and are expressed as number of green pixels and represent the average of six biological replicates per treatment ± standard deviation. Within the same row and for the specified day different letters indicate significant difference according to one-way ANOVA post-hoc Tukey’s test (p<0.05).

| Treatment | Day 6 | | Day 8 | | Day 10 | | Day 13 | | Day 15 | |
| --- | --- | --- | --- | --- | --- | --- | --- | --- | --- | --- |
| No application | 187943 ±  44101 | *b* | 202468 ±  44445 | *b* | 236701 ±  53937 | *c* | 257249 ±  72050 | *d* | 362064 ±  123515 | *c* |
| Foliar application | 198356 ±  44491 | *b* | 227917 ±  52598 | *b* | 258050 ±  44246 | *c* | 373705 ±  74638 | *cd* | 428439 ±  101159 | *c* |
| Drench application | 331285 ±  58029 | *a* | 403409 ±  62708 | *a* | 404148 ±  83744 | *b* | 601155 ±  118720 | *ab* | 686770 ±  167069 | *ab* |

**Suppl. Table 3.** Digital biomass of tomato plants cultivated under limited irrigation and subjected to treatment by PH either by spraying or drenching. Values are expressed as number of green pixels and represent the average of six biological replicates per treatment ± standard deviation. Within the same row and for the specified day different letters indicate significant difference in digital biomass, according to one-way ANOVA post-hoc Tukey’s test (p<0.05).

| Treatment | Day 6 | | Day 8 | | Day 10 | | Day 13 | | Day 15 | |
| --- | --- | --- | --- | --- | --- | --- | --- | --- | --- | --- |
| No application | 23234863 ±  8513186 | *b* | 32552528 ±  10300538 | *b* | 46577667 ±  15866094 | *b* | 82032934 ±  32296685 | *b* | 117016673 ±  53533194 | *b* |
| Foliar application | 26600180 ±  8773383 | *ab* | 40048488 ±  12400100 | *b* | 58660490 ±  16185887 | *ab* | 114478104 ±  30378481 | *b* | 157341714 ±  48413891 | *b* |
| Drench application | 39638661 ±  10594869 | *a* | 58027767 ±  11330937 | *a* | 83371143 ±  17502021 | *a* | 173594850 ±  34176174 | *a* | 236193508 ±  51170025 | *a* |

**Suppl. Table 4.** Width of the tomato plants extracted from multiple side view RGB images of the tomato plants cultivated under limited irrigation and subjected to treatment by PH either by spraying or drenching. Values are expressed as number of green pixels and represent the average of six biological replicates per treatment ± standard deviation. Within the same row and for the specified day different letters indicate significant difference according to one-way ANOVA post-hoc Tukey’s test (p<0.05).

| Treatment | Day 6 | | Day 8 | | Day 10 | | Day 13 | | Day 15 | |
| --- | --- | --- | --- | --- | --- | --- | --- | --- | --- | --- |
| No application | 582 ± 89 | *b* | 637 ± 69 | *b* | 672 ± 81 | *b* | 759 ± 86 | *c* | 851 ± 126 | *c* |
| Foliar application | 599 ± 85 | *b* | 674 ± 107 | *b* | 733 ± 100 | *b* | 861 ± 101 | *b* | 957 ± 116 | *b* |
| Drench application | 682 ± 105 | *a* | 813 ± 114 | *a* | 867 ± 144 | *a* | 1059 ± 141 | *a* | 1138 ± 149 | *a* |

**Suppl. Table 5.** Height of the tomato plants extracted from multiple side view RGB images of the tomato plants cultivated under limited irrigation and subjected to treatment by PH either by spraying or drenching. Values are expressed as number of green pixels and represent the average of six biological replicates per treatment ± standard deviation. Within the same row and for the specified day different letters indicate significant difference according to one-way ANOVA post-hoc Tukey’s test (p<0.05).

| Treatment | Day 6 | | Day 8 | | Day 10 | | Day 13 | | Day 15 | |
| --- | --- | --- | --- | --- | --- | --- | --- | --- | --- | --- |
| No application | 514 ± 69 | *b* | 639 ± 100 | *b* | 739 ± 91 | *b* | 987 ± 120 | *b* | 1166 ± 145 | *b* |
| Foliar application | 508 ± 80 | *b* | 605 ± 92 | *b* | 715 ± 100 | *b* | 987 ± 142 | *b* | 1208 ± 156 | *ab* |
| Drench application | 615 ± 22 | *a* | 724 ± 28 | *a* | 850 ± 44 | *a* | 1090 ± 68 | *a* | 1281 ± 79 | *a* |

**Suppl. Table 6.** Variation in shoot colours of tomato plants cultivated under limited irrigation and subjected to treatment by PHs either by spraying or drenching. The values for 6 most representative colour hues are shown as percentage of the shoot area (pixel counts). Values represent the average of six biological replicates per treatment ± standard deviation. Within the same row and for the specified day different letters indicate significant difference according to one-way ANOVA post-hoc Tukey’s test (p<0,05).

| Day 6 | | | | | | | | | | | | |
| --- | --- | --- | --- | --- | --- | --- | --- | --- | --- | --- | --- | --- |
| Treatment | **RGB (63,79,58)** | | **RGB (89,100,83)** | | **RGB (83,95,58)** | | **RGB (49,66,45)** | | **RGB (62,81,81)** | | **RGB (62,82,38)** | |
| No application | 24 ± 2 | *a* | 12 ± 1 | *ab* | 14 ± 6 | *a* | 24 ± 8 | *a* | 4 ± 1 | *a* | 17 ± 4 | *a* |
| Foliar application | 26 ± 5 | *a* | 10 ± 1 | *b* | 15 ± 4 | *a* | 25 ± 6 | *a* | 4 ± 1 | *a* | 16 ± 3 | *a* |
| Drench application | 29 ± 2 | *a* | 10 ± 3 | *a* | 14 ± 6 | *a* | 28 ± 5 | *a* | 3 ± 0.5 | *a* | 15 ± 5 | *a* |
| Day 8 | | | | | | | | | | | | |
| Treatment | **RGB (63,79,58)** | | **RGB (89,100,83)** | | **RGB (83,95,58)** | | **RGB (49,66,45)** | | **RGB (62,81,81)** | | **RGB (62,82,38)** | |
| No application | 26 ± 5 | *a* | 12 ± 2 | *a* | 14 ± 7 | *a* | 25 ± 9 | *a* | 4 ± 0.4 | *a* | 17 ± 3 | *a* |
| Foliar application | 28 ± 3 | *a* | 11 ± 2 | *a* | 15 ± 4 | *a* | 25 ± 4 | *a* | 4 ± 0.5 | *a* | 16 ± 3 | *ab* |
| Drench application | 28 ± 4 | *a* | 13 ± 3 | *a* | 11 ± 4 | *a* | 33 ± 8 | *a* | 4 ± 0.6 | *a* | 11 ± 5 | *b* |
| Day 10 | | | | | | | | | | | | |
| Treatment | **RGB (63,79,58)** | | **RGB (89,100,83)** | | **RGB (83,95,58)** | | **RGB (49,66,45)** | | **RGB (62,81,81)** | | **RGB (62,82,38)** | |
| No application | 27 ± 5 | *a* | 10 ± 2 | *a* | 15 ± 8 | *a* | 27 ± 9 | *b* | 3 ± 0.3 | *a* | 17 ± 3 | *a* |
| Foliar application | 29 ± 1 | *a* | 7 ± 1 | *b* | 13 ± 3 | *a* | 28 ± 4 | *ab* | 3 ± 0.3 | *a* | 17 ± 2 | *a* |
| Drench application | 31 ± 3 | *a* | 9 ± 1 | *a* | 11 ± 2 | *a* | 40 ± 6 | *a* | 3 ± 0.8 | *a* | 10 ± 5 | *b* |
| Day 13 | | | | | | | | | | | | |
| Treatment | **RGB (63,79,58)** | | **RGB (89,100,83)** | | **RGB (83,95,58)** | | **RGB (49,66,45)** | | **RGB (62,81,81)** | | **RGB (62,82,38)** | |
| No application | 26 ± 5 | *a* | 9 ± 0.3 | *a* | 17 ± 5 | *a* | 26 ± 6 | *b* | 3 ± 0.3 | *a* | 18 ± 4 | *a* |
| Foliar application | 27 ± 1 | *a* | 7 ± 3 | *a* | 15 ± 2 | *a* | 31 ± 4 | *ab* | 3 ± 0.2 | *a* | 16 ± 2 | *ab* |
| Drench application | 28 ± 3 | *a* | 9 ± 2 | *a* | 13 ± 3 | *a* | 35 ± 5 | *a* | 3 ± 0.7 | *a* | 12 ± 3 | *b* |
| Day 15 | | | | | | | | | | | | |
| Treatment | **RGB (63,79,58)** | | **RGB (89,100,83)** | | **RGB (83,95,58)** | | **RGB (49,66,45)** | | **RGB (62,81,81)** | | **RGB (62,82,38)** | |
| No application | 24 ± 4 | *a* | 11 ± 1 | *a* | 18 ± 5 | *a* | 25 ± 7 | *b* | 3 ± 0.4 | *a* | 19 ± 5 | *a* |
| Foliar application | 26 ± 2 | *a* | 9 ± 2 | *a* | 17 ± 3 | *a* | 28 ± 5 | *ab* | 3 ± 0.3 | *a* | 17 ± 2 | *a* |
| Drench application | 28 ± 4 | *a* | 10 ± 2 | *a* | 15 ± 1 | *a* | 34 ± 3 | *a* | 3 ± 0.4 | *a* | 9 ± 2 | *b* |

**Suppl. Table 7.** Photosynthetic performance of tomato plants. Photosynthetic parameters deduced from kinetic chlorophyll fluorescence imaging on whole plant level in tomato plants cultivated under limited irrigation and subjected to treatment by PH either by spraying or drenching. Minimal fluorescence in dark-adapted state (F_0_ ), maximum fluorescence in dark-adapted state (F_M_ ), maximum quantum yield of PSII photochemistry for the light-adapted state (Fv´/Fm´), the photochemical quenching coefficient that estimates the fraction of open PSII reaction centers (qP), proportion of closed PSII reaction centers (1-qP), steady-state non-photochemical quenching (NPQ) and electron transport rate (ETR) were measured using the light curve protocol for tomato plants prior and upon two times of PHs treatments. Values represent the average of six biological replicates per treatment ± standard deviation. Within the same row and for the specified day different letters indicate significant difference according to one-way ANOVA post-hoc Tukey’s test (p<0,05). Lss1, Lss2 and Lss3 represent actinic photon irradiance measurements taken at 170, 620 and 1070 µmol photons m^-2^ s^-1^ PAR values, respectively.

|  | Lss 1 | | | | | | | | | | | | | |
| --- | --- | --- | --- | --- | --- | --- | --- | --- | --- | --- | --- | --- | --- | --- |
| Treatment | **F_0_** | | **F_m_** | | **F_v_/F_m_** | | **qP** | | **1-qP** | | **NPQ** | | **ETR** | |
| No application | 75 ± 12 | *a* | 317 ± 23 | *a* | 0.77 ± 0.02 | *a* | 0.55 ± 0.32 | *a* | 0.45 ± 0.32 | *a* | 0.55 ± 0.01 | *a* | 31 ± 17 | *a* |
| Foliar application | 72 ± 9 | *a* | 293 ± 48 | *a* | 0.75 ± 0.06 | *a* | 0.57 ± 0.26 | *a* | 0.43 ± 0.26 | *a* | 0.60 ± 0.10 | *a* | 26 ± 15 | *a* |
| Drench application | 91 ± 27 | *a* | 253 ± 129 | *a* | 0.73 ± 0.08 | *a* | 0.38 ± 0.13 | *a* | 0.62 ± 0.13 | *a* | 0.57 ± 0.14 | *a* | 17 ± 6 | *a* |

|  | Lss 2 | | | | | | | | | | | | | |
| --- | --- | --- | --- | --- | --- | --- | --- | --- | --- | --- | --- | --- | --- | --- |
| Treatment | **F_0_** | | **F_m_** | | **F_v_/F_m_** | | **qP** | | **1-qP** | | **NPQ** | | **ETR** | |
| No application | 68 ± 10 | *a* | 224 ± 12 | *a* | 0.65 ± 0.03 | *a* | 0.35 ± 0.13 | *a* | 0.66 ± 0.13 | *a* | 0.91 ± 0.33 | *a* | 60 ± 41 | *a* |
| Foliar application | 66 ± 7 | *a* | 213 ± 22 | *a* | 0.66 ± 0.05 | *a* | 0.31 ± 0.27 | *a* | 0.65 ± 0.27 | *a* | 1.02 ± 0.26 | *a* | 52 ± 32 | *a* |
| Drench application | 70 ± 5 | *a* | 238 ± 48 | *a* | 0.67 ± 0.07 | *a* | 0.24 ± 0.22 | *a* | 0.76 ± 0.22 | *a* | 1.06 ± 0.13 | *a* | 36 ± 17 | *a* |

|  | Lss 3 | | | | | | | | | | | | | |
| --- | --- | --- | --- | --- | --- | --- | --- | --- | --- | --- | --- | --- | --- | --- |
| Treatment | **F_0_** | | **F_m_** | | **F_v_/F_m_** | | **qP** | | **1-qP** | | **NPQ** | | **ETR** | |
| No application | 62 ± 7 | a | 174 ± 5 | a | 0.64 ± 0.04 | a | 0.27 ± 0.18 | *a* | 0.73 ± 0.18 | *a* | 1.43 ± 0.33 | *a* | 71 ± 40 | *a* |
| Foliar application | 62 ± 6 | a | 174 ± 8 | a | 0.64 ± 0.05 | a | 0.27 ± 0.16 | *a* | 0.73 ± 0.16 | *a* | 1.45 ± 0.28 | *a* | 69 ± 34 | *a* |
| Drench application | 66 ± 5 | a | 192 ± 23 | a | 0.64 ± 0.06 | a | 0.24 ± 0.14 | *a* | 0.76 ± 0.14 | *a* | 1.46 ± 0.25 | *a* | 57 ± 29 | *a* |
